# Low resource availability drives feeding niche partitioning between wild bees and honeybees in a European city

**DOI:** 10.1002/eap.2727

**Published:** 2022-10-11

**Authors:** Joan Casanelles‐Abella, Simone Fontana, Bertrand Fournier, David Frey, Marco Moretti

**Affiliations:** ^1^ Biodiversity and Conservation Biology Swiss Federal Institute for Forest, Snow and Landscape Research WSL Birmensdorf Switzerland; ^2^ Institute of Terrestrial Ecosystems, ETH Zurich Zurich Switzerland; ^3^ Nature Conservation and Landscape Ecology University of Freiburg Freiburg Germany; ^4^ Institute of Environmental Sciences and Geography, University of Potsdam Potsdam Germany

**Keywords:** competition, intraspecific trait variability, pollinator, species interaction, urban biodiversity, urbanization

## Abstract

Cities are socioecological systems that filter and select species, therefore establishing unique species assemblages and biotic interactions. Urban ecosystems can host richer wild bee communities than highly intensified agricultural areas, specifically in resource‐rich urban green spaces such as allotments and family gardens. At the same time, urban beekeeping has boomed in many European cities, raising concerns that the fast addition of a large number of managed bees could deplete the existing floral resources, triggering competition between wild bees and honeybees. Here, we studied the interplay between resource availability and the number of honeybees at local and landscape scales and how this relationship influences wild bee diversity. We collected wild bees and honeybees in a pollination experiment using four standardized plant species with distinct floral morphologies. We performed the experiment in 23 urban gardens in the city of Zurich (Switzerland), distributed along gradients of urban and local management intensity, and measured functional traits related to resource use. At each site, we quantified the feeding niche partitioning (calculated as the average distance in the multidimensional trait space) between the wild bee community and the honeybee population. Using multilevel structural equation models (SEM), we tested direct and indirect effects of resource availability, urban beekeeping, and wild bees on the community feeding niche partitioning. We found an increase in feeding niche partitioning with increasing wild bee species richness. Moreover, feeding niche partitioning tended to increase in experimental sites with lower resource availability at the landscape scale, which had lower abundances of honeybees. However, beekeeping intensity at the local and landscape scales did not directly influence community feeding niche partitioning or wild bee species richness. In addition, wild bee species richness was positively influenced by local resource availability, whereas local honeybee abundance was positively affected by landscape resource availability. Overall, these results suggest that direct competition for resources was not a main driver of the wild bee community. Due to the key role of resource availability in maintaining a diverse bee community, our study encourages cities to monitor floral resources to better manage urban beekeeping and help support urban pollinators.

## INTRODUCTION

Many urban ecosystems harbor rich and diverse wild bee communities (Baldock et al. 2015; Casanelles‐Abella, Chauvier, et al. 2021; Theodorou et al., 2020), as opposed to that observed in other pollinator groups (Theodorou et al., 2020). At the same time many cities, such as Paris (Ropars et al., 2019), London (Stevenson et al., 2020), Munich (Renner et al., 2021) and Berlin (Lorenz & Stark, 2015), have seen a boom in urban beekeeping in recent years (Baldock, 2020; Egerer & Kowarik, 2020). Urban beekeeping is not necessarily driven only by economic reasons; it is also a leisure activity often carried out under the popular belief of helping “the bees” (Baldock, 2020) and presents both opportunities and risks for wild bee conservation (Egerer & Kowarik, 2020). When the diversity and quantity of floral resources do not grow at the same pace as the increase in bee numbers, fast and uncontrolled additions of honeybee hives within the cityscape might deplete pollen and nectar resources (Torné‐Noguera et al., 2016). Consequently, this could enhance competition between wild bees and honeybees for resources in their foraging sites (Magrach et al., 2017). In addition, major increases in the densities of honeybees over both short (Magrach et al., 2017) and long periods (Herrera, 2020) can lead to competition, regardless of the quantity of resources.

Prior studies on the effect of beekeeping on wild bees have taken place in different ecosystem types, but mostly in nonurban ecosystems. These studies have shown a disruption of interaction networks (Ropars et al., 2019), a depletion of resources (Torné‐Noguera et al., 2016), a decrease in fitness (Elbgami et al., 2014), or changes in community composition (Henry & Rodet, 2020; Herrera, 2020). It is not yet known, however, to what extent the current growth in urban beekeeping is influencing wild bee diversity.

There are several reasons why wild bees might be negatively affected by competition with honeybees, particularly concerning exploitative competition. First, honeybees are supergeneralist species, with large numbers of workers, efficient foraging behavior, large food requirements, and high mobility, which ensures high diversity of nutrients and the minimization of toxic substances (Wright et al., 2018). The honeybee foraging strategy seems to be focused on pollen quantity rather than quality, implying that honeybees visit a large variety of nutritionally different plants (Leonhardt & Blüthgen, 2012). Although social wild bees might also benefit from task division, most wild bees are solitary and females must feed themselves, build a nest and provide their larvae with specific food (Somanathan et al., 2020). Second, honeybees are managed pollinators, which are fed and kept healthy by humans; therefore, they are expected to be less subjected to natural selection than wild bees, and to overcome difficult periods more easily (drought and long‐lasting rain). Third, wild bees also have large pollen requirements for the development of their offspring (Müller et al., 2006), and many polylectic wild bees enhance their fitness by collecting pollen from many different sources (Woodard & Jha, 2017). Therefore, the depletion of pollen resources by more efficient honeybee foragers can have important negative consequences on the fitness of both adult and larval wild bees, and might increase both intraspecific and interspecific competition. Fourth, many wild bee species are expected to have relatively short foraging ranges compared with honeybees, due to mobility limitations or nutritional constraints that prevent longer flights (Woodard & Jha, 2017). Fifth, wild bee species might have restricted possibilities to switch their pollen hosts compared with honeybees, due to physiological and cognitive restrictions (Praz et al., 2008). Finally, honeybees and wildbees can transfer mutually diseases and pathogens (Klein et al., 2018).

These different processes can have a larger or smaller importance in determining the sensitivity to resource availability, as well as the niche partitioning and therefore the strength of competitive interactions between wild bees and honeybees, depending on the scale considered. Two species are more likely to coexist if they use different resources and occupy distinct niches (limiting similarity; Macarthur & Levins, 1967). Therefore, in this study we defined niche partitioning as the tendency of two species or individuals to occupy different ecological niches (i.e., use distinct resources or use the same resources but at distinct times). Niche overlap is the opposite of niche partitioning (i.e., the degree to which two organisms share the same ecological niche). Ultimately, differences among traits specific to wild bees and honeybees that are related to resource use will determine the niche overlap and therefore the degree of competition.

At the same time, resource availability influences to what extent individuals share the same resources (i.e., niche partitioning) and modulates the strength of competitive interactions, with the traits of the individuals determining the scale at which these processes operate. Wild bees exhibit a large spectrum of traits related, for example, to foraging and dispersal. Some large bees (e.g., *Bombus* spp.) might have a large foraging range and therefore be less sensitive to processes affecting resource availability at smaller spatial scales (Hennig & Ghazoul, 2011). However, many wild bee species have consistently been found to be promoted by local resource availability (Baldock et al., 2019; Theodorou et al., 2020; please also refer to Baldock, 2020). In particular, enhanced resource availability, e.g., due to reduced management, has been shown to affect wild bee community composition (Lerman et al., 2018), increase bee diversity (Braaker et al., 2017), expand the duration of bumblebees' phenology (Stelzer et al., 2010), and improve the robustness of plant–pollinator interactions (Baldock et al., 2019).

Although evidence is still scarce, competition is thought to be an important driver of wild bee community structure, for instance, by modulating floral fidelity and reproductive success (Brosi & Briggs, 2013; Fründ et al., 2013). The strength of competitive interactions is also affected by the resource availability at species‐specific spatial scales. In the context of urban beekeeping, honeybees can affect wild bees at both local (i.e., local densities; Renner et al., 2021) and landscape scales (i.e., number of apiaries; Ropars et al., 2019), as local honeybee densities are usually correlated with the number of apiaries in the surrounding areas (Steffan‐Dewenter & Tscharntke, 1999). Conversely, because honeybees are managed pollinators with foraging features distinct from those of wild bee species, honeybee densities might be determined mostly by the distribution of their apiaries (Steffan‐Dewenter & Tscharntke, 1999). Furthermore, honeybee morphologic, physiologic, and behavioral traits might enable them to easily switch foraging sites, and therefore be less sensitive to the processes affecting resource availability at different landscape scales such as habitat loss, fragmentation, disturbance, or stress (Hennig & Ghazoul, 2011).

Novel insights into these complex relationships might be gained through trait‐based approaches, which are increasingly considered a promising way to pursue the specific goal of understanding biotic interactions, including competition (McGill et al., 2006). For example, functional traits, that is, the phenotypic attributes of an individual that determine its fitness (Violle et al., 2007), have been successfully used to predict species interactions in plant–pollinator networks (Pichler et al., 2020). Indeed, functional traits can be linked directly to the mechanisms of species coexistence, which include resource or niche partitioning (Chesson, 2003). We note that niche partitioning is multidimensional, involving several traits related to resource use. The concept of a multidimensional niche space (Hutchinsonian hypervolume; Hutchinson, 1957) and the theory that competition for resources sets a limit to the similarity of coexisting species (Macarthur & Levins, 1967) are not novel. The same is true for the use of multiple traits to quantify resource partitioning among animal species (e.g., Gautier‐Hion et al., 1985). However, in the last decades renewed interest was stimulated by the development of a plethora of trait databases and multidimensional metrics that potentially quantify niche partitioning with unprecedented accuracy, for example by considering intraspecific trait variation (e.g., Albert et al., 2012; Cianciaruso et al., 2009; Fontana et al., 2021; Pavoine & Izsák, 2014). This was an important step, as stabilizing mechanisms of coexistence, such as resource partitioning, explicitly affect the relative magnitude of both inter‐ and intraspecific competitive interactions (Chesson, 2000). Therefore, several studies have shown the importance of intraspecific trait variation across multiple traits in determining competitive interactions, as well as population and community dynamics (e.g., Bolnick et al., 2011; Des Roches et al., 2018; Fontana et al., 2019).

**FIGURE 1:**
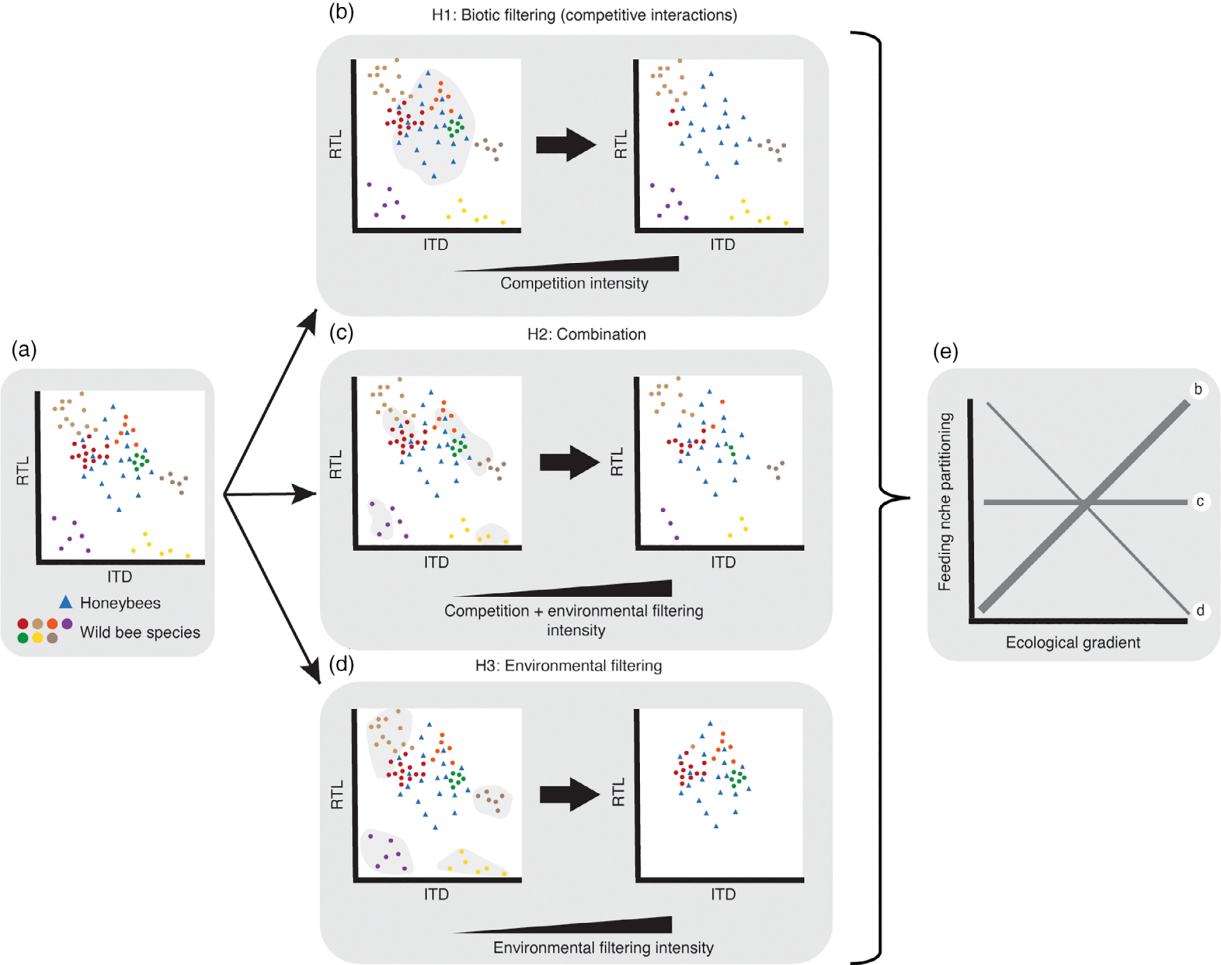
Illustration of the functional metric and hypotheses regarding the drivers of wild bee community structure. (a) A hypothetical bee community, composed of seven wild bee species (colored circles) and honeybees (blue triangles), depicted in a trait space defined by two traits: intertegular distance (ITD, *x*‐axis) and relative tongue length (RTL, *y*‐axis). Three hypotheses are presented regarding the influence of competition (biotic filtering) and environmental filtering in shaping the functional composition of a bee community (b–d) and the feeding niche partitioning between wild bees and honeybees (e). In (b), competition intensity is assumed to be the main driver shaping the wild bee community. Competition intensity can increase as a result of higher beekeeping intensity (larger number of honeybee individuals in a site or hives in the surrounding landscape) and/or lower resource availability. Therefore, with increasing competition intensity, wild bee species that are functionally similar to honeybees (dots inside the gray patch) are expected to be outcompeted and removed from the community due to (excessive) niche overlap. In the opposite scenario (d), a certain environmental gradient (e.g., urbanization intensity) is assumed to be the main driver shaping the wild bee community. Environmental filtering intensity represents environmental drivers that filter traits without necessarily influencing biotic interactions (competition) directly, for example, temperature, habitat loss, and fragmentation. Therefore, with increasing environmental filtering intensity, wild bee species functionally dissimilar to honeybees (dots inside the gray patches) are filtered out, and the best adapted phenotypes under these types of environmental conditions (i.e., those similar to honeybees) dominate the community. In (c), an intermediate scenario is shown, in which both biotic interactions and environment are expected to simultaneously shape the wild bee community. In (e), the changes in the community niche partitioning (defined as the mean pairwise distance of all wild bee individuals with all honeybee individuals) under the different hypotheses are represented in a simplified plot. Thicker lines indicate a major influence of biotic interactions (b), whereas thinner lines indicate that environmental conditions have a larger impact (d). As the two processes are expected to drive feeding niche partitioning in opposite directions, no change may be observed along environmental gradients (c, flat line). Note that, for simplicity, only two traits are depicted in (a–d), whereas only three linear lines are plotted in (e) even though other relationships could occur.

In this study, we aimed to investigate the role of the interplay between urban beekeeping and resource availability at different spatial scales and how this relationship influences wild bee diversity in urban ecosystems. To do so, we measured functional traits related to the resource use of individual bee specimens collected in a pollination experiment set in domestic gardens distributed following gradients of beekeeping intensity and resource availability at different scales. We quantified feeding niche partitioning between the wild bee community and the honeybee population using functional traits related to feeding behavior and preferences. We tested the following hypotheses (please refer to also Figure 1): (1) higher urban intensity at the landscape scale negatively affects the number of wild bee species; (2) higher plant species richness at the local scale positively affects the number of wild bee species; (3) the number of wild bee species is negatively related to the beekeeping intensity at both the local (number of individuals at the sampling site) and landscape scale (number of hives around sampling sites); (4) if the pattern in (3) is driven by opposite responses to environmental conditions (e.g., habitat loss), with many wild bee species being less adapted than honeybees to the fragmented habitat in highly urbanized areas, we expect wild bees to be functionally similar to honeybees (i.e., with similar traits) to survive where honeybees are abundant (feeding niche overlap increases while feeding niche partitioning decreases; Figure 1d). Conversely, if this pattern is driven by true competition for flower resources, we expect the wild bee community to be on average less similar to honeybees, and therefore show increased feeding niche partitioning (lower niche overlap, because wild bees that are too similar to honeybees are outcompeted by the latter; Figure 1b).

## METHODS

### Study design

The study was conducted in the city of Zurich, Switzerland (47°22′0″ N, 8°33′0″ E), located in the Swiss Central plateau. Zurich is the largest urban area in Switzerland, covering 92 km^2^ and with a population of ~400,000 inhabitants within the municipality. It contains many different types of green areas, which cover more than 43% of the city (Grün Stadt Zürich, unpubl. data). We sampled 23 urban gardens in the city of Zurich along two independent local‐scale and landscape‐scale gradients. The two gradients were chosen to separate the effects of local resource availability and potential foraging habitat at (larger) landscape spatial scales (please refer to Appendix S1: Table S1). Specifically, we selected the gardens to vary independently in their flower species richness and in the proportional amount of impermeable surface in the surrounding landscape. All gardens were mostly open regarding vegetation, with at least 7 h of daily sun exposure.

### Experimental setup

We set up an array of 19 pots containing four phytometer plant species in the center of each garden (please refer to Appendix S1: Figure S1). Each pot contained one plant of the following insect‐pollinated plant species, which differed in nectar accessibility: *Daucus carota* L. (wild carrot, Apiaceae; five pots), *Raphanus sativus* L. (radish, Brassicaceae; six pots), *Onobrychis viciifolia* Scop. (common sainfoin, Fabaceae; five pots), and *Symphytum officinale* L. (common comfrey, Boraginaceae; three pots). These species were selected based on their expected variation in flower visitor specificity because of their differences in floral type. They were also selected because of their large number of flowers (>100) or inflorescences, similar height (~30–100 cm) and long and overlapping flowering time (May through August). Please refer to Appendix S1: Section S1 for details on how the plants were acquired, sown, and grown.

### Bee specimens sampling

We collected wild bee and honeybee individuals on each of the four plant species during their peak flowering time between 15 June and 20 July 2016. In each garden, two or three people simultaneously collected bees for a full and consecutive 9 h between 9:00 AM and 6:00 PM under sunny weather conditions and wind speed <2 m/s, at least 3 days in each garden (3 days in eight gardens, 4 days in 11 gardens and 5 days in four gardens, please refer to Appendix S1: Table S1). To achieve this, we recruited 37 volunteers and trained them to sample up to nine gardens in parallel on the same day. Volunteers were randomly allocated to gardens for each sampling round, but no volunteer could work twice in the same garden. The volunteers collected bees that had landed on an open flower. Each bee was transferred individually to an 8 ml glass tube, which was labeled with the respective phytometer plant and capturing time window. The bees were kept at −20°C and were determined by taxonomic experts. The vast majority of specimens were identified to the species level. *Bombus terrestris* and *B. lucorum* were considered a single species complex due to difficulty distinguishing the workers of these two species (Falk, 2015). Similarly, we also aggregated *Halictus simplex*, *H. compressus*, and *H. langobardicus*. A minority of individuals that could not be assigned unambiguously to any species or species complex was identified to the genus level (i.e., *Bombus* sp. with three individuals, *Hylaeus* sp. with nine individuals, *Sphecodes niger* with two individuals).

### Bee traits

We selected six ecologically relevant functional traits related to resource exploitation and potentially influencing feeding niche partitioning with honeybees and therefore competitive interactions. Specifically, we considered the following traits (Appendix S1: Table S2): (1) intertegular distance (ITD); (2) relative tongue length; (3) feeding specialization; (4) phenology start; (5) phenology end; and (6) daytime activity. Additional information on the ecological relevance of the selected traits can be found in the Appendix S1: Section S2. We used two levels of measurement for the traits. Individual‐level trait measurements were taken for ITD, tongue length, and daily activity, species‐level measurements for the remaining traits were extracted from the European trait database (compiler: Stuart Roberts; pollinator loss module of the EU‐FP6 ALARM‐project). Please refer to Appendix S1: Section S2 for detailed information on the individual‐level measurements.

### Environmental variables

#### Resource availability at the local scale

We used two proxies to assess resource availability at the local scale, specifically plant species richness and garden management. We computed plant species richness as the total number of plant species sampled in each study site, including both spontaneous and cultivated plant species (and excluding the four phytometer species used for the experiment). We sampled plant species using floristic inventories, as described by Frey and Moretti (2019). To infer garden management intensity, we used an index similar to that applied by Smith et al. (2006), based on a 26‐question questionnaire about the physical and chemical management practices of each garden (please refer to Tresch et al., 2019 for details).

#### Resource availability at the landscape scale

We inferred resource availability at the landscape scale by calculating the amount of different green and impervious (gray) land‐cover types at multiple spatial scales. Impervious land covers, both build and paved, are a common proxy of urban intensity and negatively related to the resource availability and the amount of available habitat (Harrison & Winfree, 2015). Conversely, green land covers, which include different compositions and configurations of the vegetation, are positively related to resource availability. We considered multiple spatial scales because the perception of organisms to landscape‐scale features varies according to their traits (Tscharntke et al., 2012). The combination of several small (i.e., 50–250 m) and one relatively large (500 m) spatial scales has been found to adequately capture the variation in species composition and ecosystem processes in similar studies in the same city (Braaker et al., 2014; Frey et al., 2018; Hennig & Ghazoul, 2011). More details can be found in Appendix S1: Section S3.

#### Beekeeping intensity

At the local scale, we used the number of honeybee individuals sampled in the study sites to quantify beekeeping intensity. At the landscape scale, we calculated the number of honeybee hives within a 100, 250, 500, 1000, and 2000 m radii (following Leguizamón et al., 2021; Ropars et al., 2019) as a surrogate for the density of potential competitors in the surrounding landscape. Information on the distribution of honeybee hives was obtained from the veterinary office of the canton of Zurich for the year 2016.

### Statistical analyses

All analyses were carried out in R environment version 4.0.2 (R Core Team, 2021) with the help of RStudio version 1.4.1106 (RStudio Team, 2020).

#### Calculation of feeding niche partitioning

To quantify the degree of feeding niche partitioning between the wild bee community and the honeybee population, for each site we calculated the average pairwise Euclidean distance between wild bee and honeybee individuals in the multidimensional trait space defined by six feeding‐related functional traits (ITD, relative tongue length, feeding specialization, phenology start, phenology end, daytime activity; please refer to the “*Bee traits*” section and Appendix S1: Table S2). No dimensionality reduction or ordination technique was applied, so that the position (coordinates) of each individual in a six‐dimensional trait space was objectively determined by all six abovementioned traits (defined as axes). Prior to the niche partitioning calculation, all traits were scaled by subtracting the mean and dividing by the standard deviation, to ensure that they had the same unit. Please note that distances between different wild bee individuals and between different honeybee individuals were not included in the calculation of the average distance (Appendix S1: Figure S2). We excluded from the calculations and feeding niche partitioning analyses the individuals from which no traits could be measured at the individual level (i.e., individuals damaged) or whose species‐level trait were missing (*Anthidium strigatum*, one individual; *Bombus vestalis*, two individuals; *Lasioglossum glabriusculum*, one individual; *Osmia adunca*, two individuals*; Osmia leucomelana*, five individuals; *Sphecodes niger*, two individuals; *Sphecodes* sp., two individuals).

#### Structural equation models (SEM)

We assessed direct and indirect effects on community niche partitioning of the proxies for resource availability and for the number of potential competitors (i.e., both wild bees and honeybees) at local and landscape scales using multilevel structural equation modeling, implemented in the *piecewiseSEM* package v.2.1.2 in R (Lefcheck, 2016), following Shipley (2016) and Tresch et al. (2019). We used linear models as composite SEMs. We performed basis set constructions, goodness‐of‐fit tests, and parameter estimations according to the corrected Akaike information criterion (AIC_c_), the Bayesian information criterion (BIC), and Fisher's C statistic (*p* < 0.05; Shipley, 2016).

Our SEM always included four models, using community niche partitioning, the wild bee species richness, the plant species richness, and the number of honeybee individuals as response variables. To test for the interplay between resource availability and beekeeping intensity at the local and landscape scales, the models describing the community niche partitioning and wild bee species richness always included four interaction pathways with variables representing resource availability and beekeeping intensity at local and landscape scales. In addition, for feeding niche partitioning we added a path with the wild bee species richness.

We computed pairwise correlation coefficients among explanatory variables prior to analyses, and excluded variables with coefficients more than 0.7 (Appendix S1: Figures S4 and S5). Furthermore, missing paths in the SEM were checked with Shipley's d‐separation test, which enables to modify the piecewise SEM model to account for missing or incomplete pathways for each individual model that is part of the piecewise SEM model (Shipley, 2013).

The final SEM model included the following terms: (1) for the feeding niche partitioning we used the wild bee species richness, the plant species richness, the number of honeybee individuals, the number of honeybee hives at 500 m and the amount of green areas in 500 m; (2) for the wild bee species richness we used the plant species richness, the number of honeybee individuals, the number of honeybee hives at 500 m and the amount of green areas in 100 m; (3) for the plant species richness, we included the management intensity; (4) for the number of honeybee individuals, we used the plant species richness and the amount of green areas in 500 m (please refer to also Appendix S1: Table S3). Sampling effort, that is, the time each field worker monitored flowers of phytometer species, could not be standardized perfectly, so we included sampling time as an offset term in the models (Korner‐Nievergelt et al., 2015). Finally, we checked model assumptions, as well as potential spatial autocorrelation patterns in the response variables and the model residuals, by means of Moran's I autocorrelation.

## RESULTS

In total, we collected 3248 wild bee individuals from 13 genera and 63 species, as well as 577 honeybee individuals. The families Halictidae and Colletidae were the most represented, with 1625 and 1074 individuals, respectively. Concerning the functional traits, most individuals belonged to polylectic species (3201 individuals of 54 species, Appendix S1: Figure S3), whereas we found only eight oligolectic species with a total number of 33 individuals. The average phenology start occurred in February (7.17 ± 2.46 weeks from the beginning of the year) and the average phenology end occurred in May at 19.14 ± 2.66 weeks from the beginning of the year (Appendix S1: Figure S3). Average ITD ranged from 0.68 in *Hylaeus taeniolatus* to 5.74 in *Bombus terrestris* (mean ± SD = 1.49 ± 0.87 cm; Appendix S1: Figure S3) and average relative tongue length ranged from 0.59 cm in *Hylaeus pictipes* to 4.12 cm in *Bombus hortorum* (1.37 ± 0.53; Appendix S1: Figure S3). Finally, we found feeding specialization (lecty status) and phenology start and end of the wild bees to be the traits most influential on the feeding niche partitioning (functional dissimilarity) between wild bee and honeybee individuals (Appendix S1: Figure S3).

**FIGURE 2:**
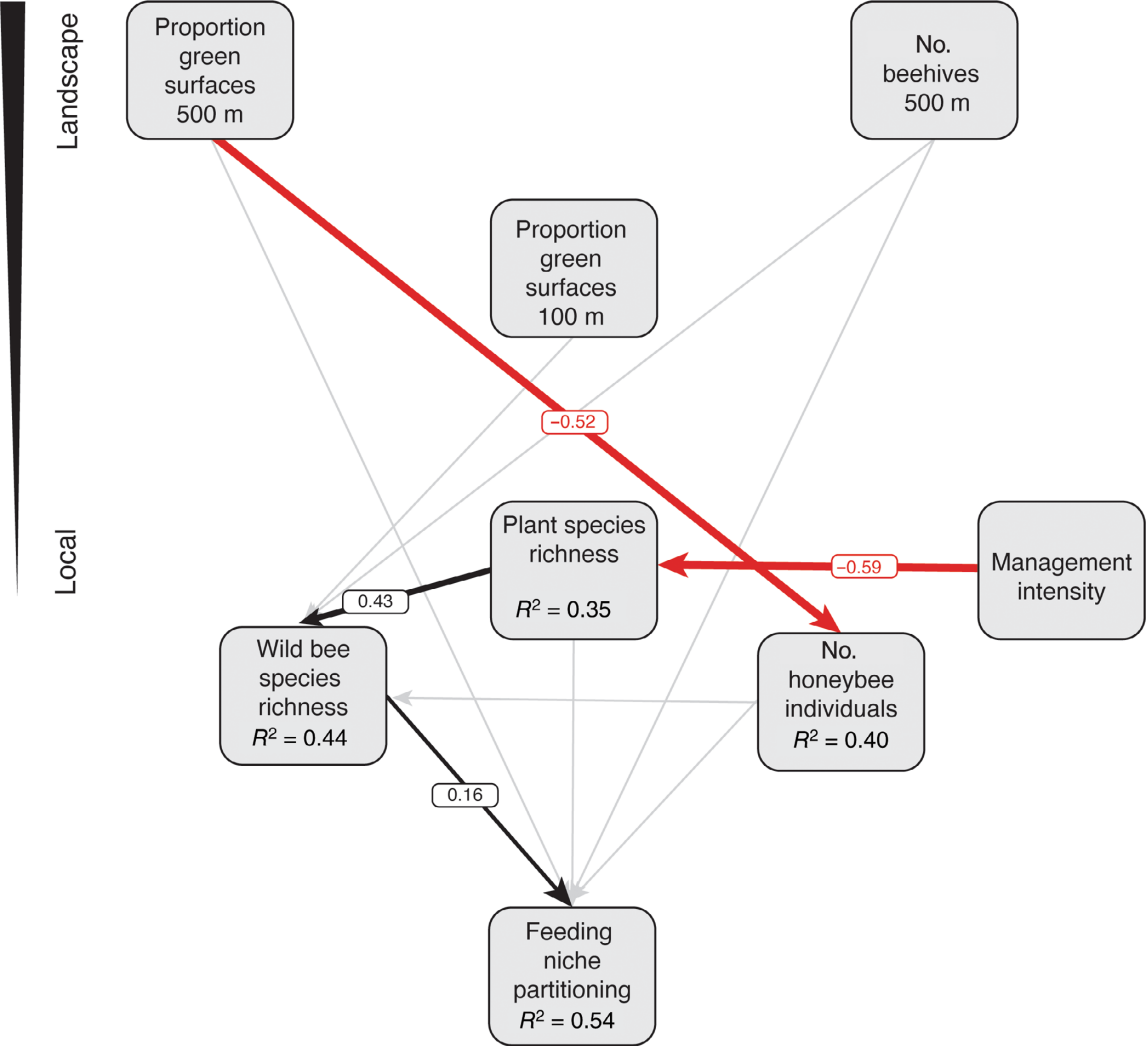
Final structural equation model (SEM). The SEM model shows the direct and indirect effects of the proxies for resource availability and beekeeping intensity at the landscape and local scale on wild bee diversity proxies, that is, wild bee species richness and the feeding niche partitioning (i.e., the mean pairwise distances between wild bee and honeybee individuals in a given site). The SEM model also includes two models explaining the factors shaping the plant species richness at the local site (proxy of resource availability at the local scale) and the number of honeybee individuals (proxy of beekeeping intensity at the local scale). The thickness of paths has been scaled based on the magnitude of the standardized regression coefficient. Numbers show standardized path coefficients for significant pathways. Positive paths are depicted in black, negative in red, and nonsignificant (*p* > 0.05) in gray. For each response variable, the *R* ^2^ is provided inside the box. AIC_c_ = 778.81, Fisher's C = 27.72, *p*‐value = 0.116. AIC_c_, corrected Akaike information criterion.

**FIGURE 3:**
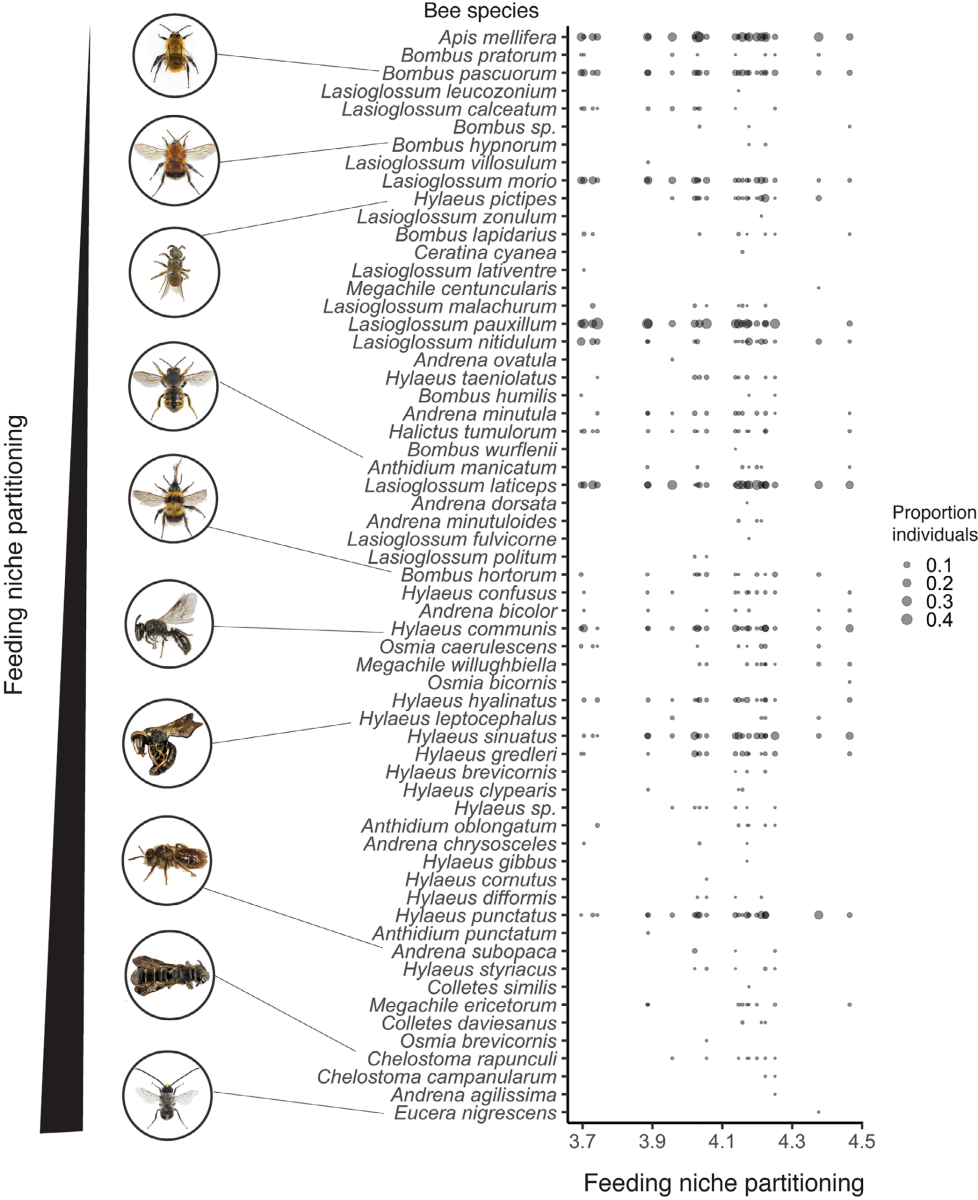
Changes in feeding niche partitioning. Wild bee species composition in relation to feeding niche partitioning value (i.e., the mean pairwise distances between wild bee and honeybee individuals in a given site) at each site. Wild bee species are sorted according to their functional dissimilarity to honeybees, with functionally similar species on the top and functionally dissimilar species on the bottom. The size of each dot represents the proportion of individuals sampled at a given site. Images retrieved from: https://www.flickr.com/people/usgsbiml/.

**FIGURE 4:**
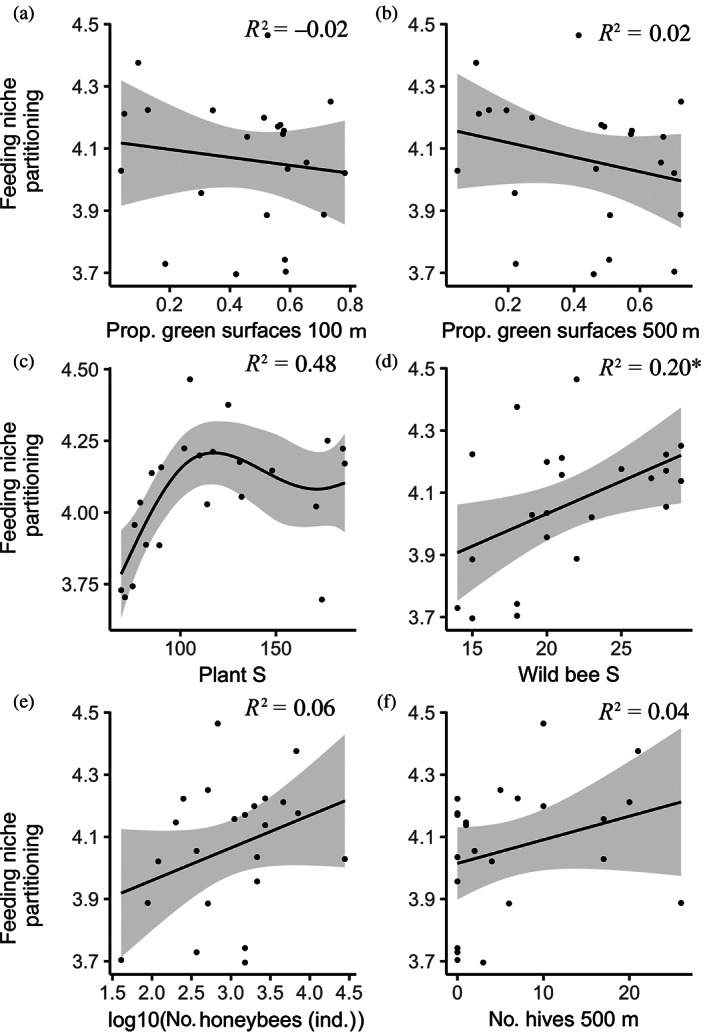
(a, b, e, f) Linear models and (c, d) generalized additive models (GAM) with the adjusted *R* ^2^ between feeding niche partitioning and resource availability at the landscape scale using as proxy the proportion of green areas at 100 m (a) and 500 m (b); resource availability at the local scale, using as a proxy the plant species richness (c); wild bee species richness (d), and urban beekeeping at the local scale, using as a proxy. The number of honeybee individuals (e); and landscape scale, using as a proxy the number of honeybee hives at 500 m (f). Smooth terms in GAMs are calculated using cubic regression splines. Gray bands indicate 95% confidence intervals. Dots represent the feeding niche partitioning between the wild bee community and the honeybee population at each of the 23 studied gardens. Please refer to Appendix S1: Figures S7–S11 for additional plots depicting the community composition along gradients of beekeeping intensity and resource availability at local and landscape scales. Significance values: *0.01 < *p* < 0.05.

**FIGURE 5:**
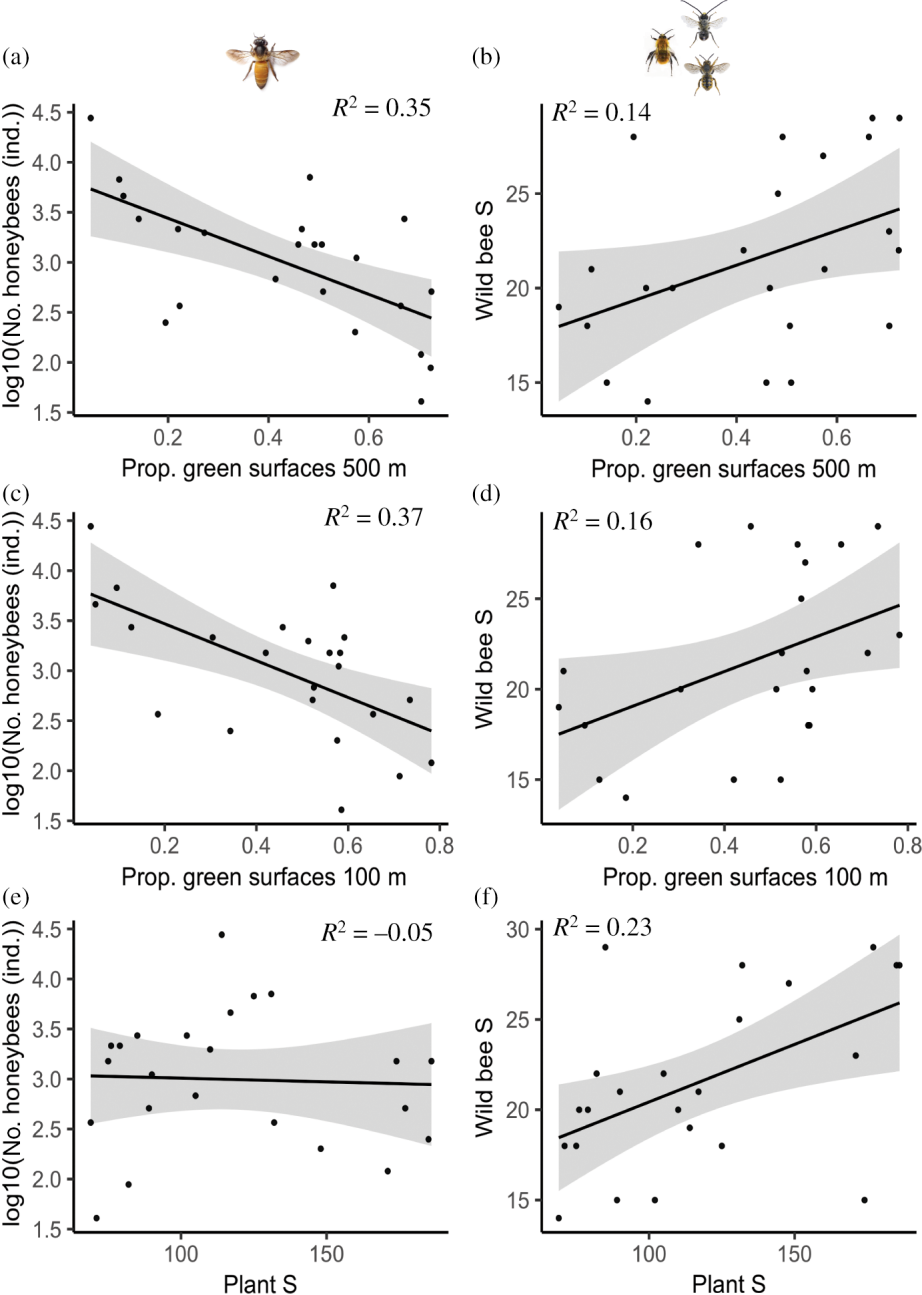
Influence of landscape and local resource availability on the number of honeybee individuals and wild bee species richness in the 23 studied gardens. Linear models depicting the relationship between the number of honeybees (a, c, e) and the wild bee species richness (b, d, f) with the proportion of green surfaces in a 500 m radius (a, b) and 100 m radius (c, d), and the local plant species richness (e, f). For each linear model, the adjusted *R* ^2^ is provided. Gray bands indicate the 95% confidence intervals. Black dots represent the study gardens. Dots represent each of the 23 studied gardens. S, species richness.

We used the multilevel SEM (Figure 2) to test both direct and indirect effects of resource availability and competitor density at both the local and landscape scale on wild bee diversity, particularly concerning feeding niche partitioning (i.e., mean pairwise distance between wild bees and honeybees). The selected SEM had no missing relationships between unconnected variables (AIC_c_ = 778.81, Fisher's C = 27.72, *p*‐value = 0.116). The SEM explained 53.77% of the variation in the niche partitioning. The feeding niche partitioning at a site increased with increasing wild bee species richness (Figures 2 and 3), specifically through the addition of wild bee species that are highly dissimilar to honeybees (last eight species in Figure 3). Furthermore, we detected changes in relative tongue length, daily activity, feeding specialization, and phenology start and end of the wild bee community with increasing values of feeding niche partitioning (Appendix S1: Figure S12b–f). In particular, with increasing feeding niche partitioning, (1) the relative tongue length of the wild bees tended to decrease (Appendix S1: Figure S12b), (2) both the start and end of the wild bee phenology was delayed (Appendix S1: Figure S12e–f), (3) the daily activity of the wild bees increased (Appendix S1: Figure S12c), and (4) the proportion of oligolectic wild bees increased (Appendix S1: Figure S12b). Nonetheless, oligolectic bees represented in all cases a small proportion of the total wild bee community (i.e., eight species and 33 individuals). Conversely, feeding niche partitioning was not affected by the proxies for resource availability at the landscape scale (Figures 4 and 5b,c). Similarly, feeding niche partitioning was not affected by proxies for beekeeping intensity at any scale (Figures 4 and 5), although we observed a nonsignificant positive relationship with the number of honeybee individuals and the number of honeybee hives in 500 m (Figure 5f,g; Appendix S1: Figures S8 and S11). Finally, feeding niche partitioning was not affected by the local plant species richness when linear models were implemented in the SEM (Figure 2). However, we detected a nonlinear relationship between feeding niche partitioning and local plant species richness (Figure 4c). For consistency in the analyses, this relationship was modeled in the SEM as a linear model.

Wild bee species richness was not significantly affected by beekeeping intensity at any spatial scales (Figure 2; Appendix S1: Figures S8b and S11b). Nonetheless, wild bee community composition was affected by beekeeping intensity (Appendix S1: Figures S8 and S11), with several species disappearing when both the number of honeybee individuals at the site (Appendix S1: Figure S8), and the number of the honeybee hives in 500 m were high (Appendix S1: Figure S11).

**FIGURE 6:**
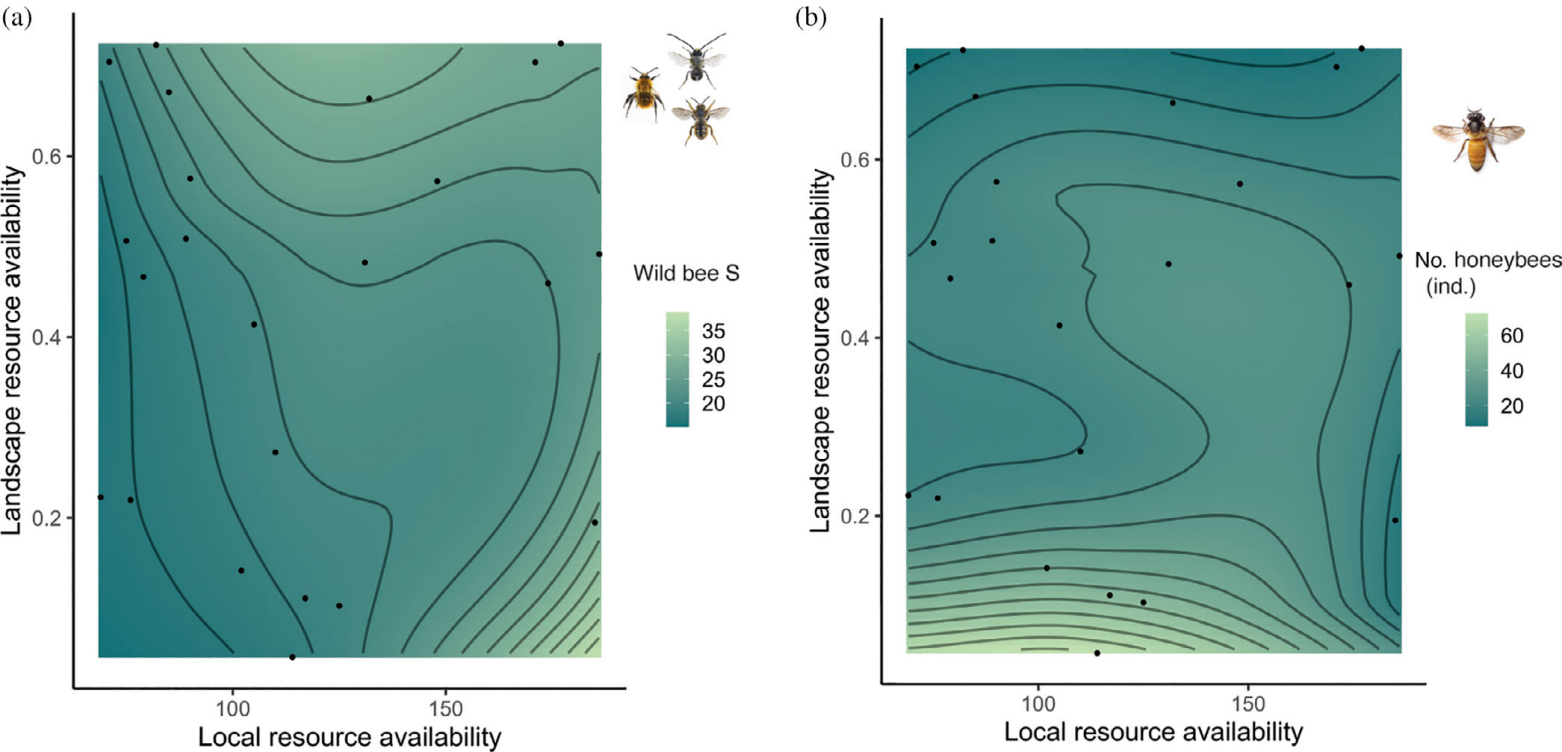
Contour plots of the predicted number of wild bee species (a) and the number of honeybees (b) with respect to resource availability at the local and landscape scale, showing that wild bee species richness and honeybee abundances are influenced by resource availability at different spatial scales (local and landscape scale, respectively). Contour plots are based on a locally estimated scatterplot smoothing (LOESS) model on the plant species richness (local resource availability) and proportion of green surfaces in a 500 m radius (landscape resource availability). Dots represent each of the 23 studied gardens.

We found wild bee species richness and honeybee abundance to be affected by resource availability at different spatial scales (Figures 4 and 5). Wild bee species richness was positively influenced by local plant species richness (Figures 2 and 5f), and, although not significant, slightly by resource availability at the landscape scale (Figure 5b,d). Predicted wild bee species richness indicated maximum wild bee species richness when resource availability at the local scale (i.e., plant species richness) was also maximal (Figure 6a), and to a minor extent, at intermediate levels of resource availability at the local scale when resource availability at the landscape scale was maximal (Figure 6a). By contrast, the number of honeybees at the site covaried negatively only with resource availability at the landscape scale (Figures 2, 5 and 6). Predicted numbers of honeybee individuals showed that honeybees concentrated at the local sites when resource availability at the landscape scale was low (Figure 6b). Furthermore, plant species richness at the site was negatively affected by management intensity (Figure 2). Finally, model coefficients and *p*‐values can be found in Table 1, alternative models in Appendix S1: Table S3, correlations among responses and predictors in Appendix S1: Figures S4 and S5, and tests of model assumptions in Appendix S1: Figure S6.

**TABLE 1:** Multilevel SEM of direct and indirect effects of the landscape factors on the number of honeybee individuals, wild bee species richness, plant species richness, and feeding niche partitioning, that is, functional dissimilarity to honeybees (AIC_c_ = 778.81, Fisher's C = 27.72, *p*‐value = 0.116).

Response	*R* ^2^	Predictor	Estimate	*p*
Feeding niche partitioning	0.54	**Wild bee species richness**	0.163 ± 0.047	*
		Amount green surface in a 500 m radius	0.121 ± 0.049	
		Plant species richness	0.011 ± 0.052	
		No. honeybee individuals	−0.052 ± 0.057	
		No. honeybee hives in a 500 m radius	0.047 ± 0.046	
Plant species richness	0.35	**Management intensity**	−0.597 ± 0.176	**
No. honeybee individuals	0.4	**Amount green surface in a 500 m radius**	−0.522 ± 0.171	**
		Plant species richness	−0.022 ± 0.171	
Wild bee species richness	0.44	**Plant species richness**	0.429 ± 0.178	*
		**Amount green surface in a 100 m radius**	0.529 ± 0.229	*
		No. honeybee individuals	0.158 ± 0.229	
		No. honeybee hives in a 500 m radius	−0.071 ± 0.187	

*Note*: For each response variable, the *R* ^2^ is provided. Significant paths are highlighted in bold. **p < 0.01; *p < 0.05.

Abbreviations: AIC_c_, corrected Akaike information criterion; SEM, structural equation models.

## DISCUSSION

Our results indicate a major effect of resource availability (bottom up) at different spatial scales in shaping urban wild bee diversity. In urban ecosystems, the management of urban greenspaces (e.g., gardening and selecting certain flower types) has a strong effect on resource availability through changes in plant diversity patterns (species, traits), which subsequently affect pollinators (e.g., Garbuzov & Ratnieks, 2014; Theodorou et al., 2020). Many wild bee species have a certain degree of patch fidelity (Ogilvie & Thomson, 2016), which could explain the importance of local resource availability in our study in enhancing wild bee species richness and, consequently, feeding niche partitioning. This was mainly due to the occurrence of wild bee species functionally distinct from honeybees, which possess traits, mainly those related to mobility or feeding specialization (please refer to also Fournier et al., 2020), that constrain their distribution within urban ecosystems more strongly than honeybees and other wild bee species that are functionally similar to honeybees. More specifically, the additional wild bee species that increase both species richness and feeding niche partitioning generally have a late phenology and are active later during the day, are oligolectic and possess a short tongue relative to their body size (Appendix S1: Figure S12).

We found that resource availability at the landscape scale was also the main driver of local honeybee abundance. In agroecosystems, honeybees have been found to adapt their foraging patterns and densities to the most abundant floral resource (Bänsch et al., 2020; Leonhardt & Blüthgen, 2012). In contrast, there is still no clear evidence regarding how resource availability affects honeybee abundance in urban ecosystems. It has been assumed that the densities of honeybees in urban ecosystems are more influenced by the distribution of the apiaries than by resource availability (Hennig & Ghazoul, 2011), due to honeybee foraging and movement traits (Goulson, 2003). In addition, most urban ecology studies do not include honeybees in their analyses (e.g., Braaker et al., 2014, 2017; Fortel et al., 2014) and the few that have included them found no effect of resource availability at any spatial scale (e.g., Wilson & Jamieson, 2019). Nonetheless, we found a clear effect of resource availability at the landscape scale on honeybee abundance, indicating that honeybees tend to concentrate in sites with few suitable habitats in the surrounding area. In a study in Brighton, UK, Garbuzov et al. (2014) reported that honeybees foraged locally within urban areas and with relatively small activity ranges. Despite being broad generalist and mobile species, honeybees have marked foraging economics, and therefore select foraging sites only if they are of sufficient quality (Garbuzov et al., 2014; Hennig & Ghazoul, 2011). In intensified urban landscapes, flower‐rich allotment gardens might represent especially valuable foraging sites, prompting honeybees to concentrate there.

Although not significant, we detected an decrease in niche partitioning with lower resource availability at the landscape scale. The decrease in feeding niche partitioning with an increasing area of green surfaces within a 500 m radius, and therefore with lower resource availability at the landscape scale, could be a consequence of stronger competition for fewer resources, that is, the phenotypes most similar to honeybees are outcompeted. If, by contrast, environmental filtering were to be the main driver, through factors not directly linked to competition, the phenotypes most similar to honeybees would be expected to survive/increase because honeybees are well adapted to these conditions, resulting in reduced niche partitioning (Figure 1c). Urban ecosystems represent pronounced environmental gradients, for example, in stress, disturbance, and habitat, which can exert a strong filtering effect, resulting in simplified species assemblages of few very dominant species in the city areas with harsh environmental conditions (Casanelles‐Abella, Chauvier, et al., 2021; Shochat et al., 2010). Shochat et al. (2010) proposed that competitive interactions with nonnative synanthropic species are a main driver of biodiversity loss, using examples of birds and spiders. This has been confirmed at the global scale, at which competitive interactions have been found to limit the occurrence of several bird species in cities worldwide (Martin & Bonier, 2018), yet it has been observed that the effect becomes less clear at smaller (within‐city) spatial scales (Planillo et al., 2021).

In any case, our results have to be interpreted with caution. First, the effect of decreasing green surface area on niche partitioning was not significant and not very pronounced (Figures 2 and 3c). Second, we did not find a direct effect of beekeeping intensity proxies at either the local or landscape scale on feeding niche partitioning nor on wild bee species richness. Still, considering that beekeeping in Zurich has increased since this experiment was carried out (Casanelles‐Abella & Moretti, 2022), our results could be interpreted as a warning signal of the future consequences of uncontrolled increases in urban beekeeping. A possible reason for the lack of a negative relationship between beekeeping intensity and feeding niche partitioning is that beekeeping intensity for the focal year, together with resource availability at the relevant spatial scales, created the conditions to enable wild bee and honeybee coexistence. Moreover, although the study sites, that is, the urban gardens, varied in plant richness, they represent an urban land use typically rich in flowers and floral resources (e.g., Baldock et al., 2019; Tew et al., 2021), which might allow coexistence between wild bees and honeybees to a greater extent than in other urban land uses and urban habitats. Finally, urban wild bee communities represent an already filtered subset of the regional wild bee species pool, with features that make them better adapted for surviving and thriving in urban ecosystems (Fournier et al., 2020). On the one hand, functionally similar wild bee species that are likely to have resource‐use overlap with honeybees (e.g., several *Bombus* spp.) might be able to switch to alternative plants (Briggs & Brosi, 2017) or move to other foraging patches, although they were also constrained by nutritional requirements (Wignall et al., 2020) and their specific feeding behavior (e.g., patch fidelity; Ogilvie & Thomson, 2016). On the other hand, functionally dissimilar wild bee species may have limited or no resource overlap with honeybees, making their occurrence more sensitive to features other than beekeeping intensity, particularly at the local scale (Casanelles‐Abella, Chauvier, et al., 2021).

Although we found no significant influence of beekeeping intensity on feeding niche partitioning, this does not necessarily mean that urban beekeeping does not pose any risks to wild bees. Urban ecosystems are especially dynamic relative to other ecosystems, in part because individual or collective decisions and actions can occur rapidly and propagate to the whole city (Alberti, 2015). In our case, urban beekeeping is a relatively new activity in cities, but it has undergone fast and unregulated growth in recent years. For example, in Zurich the number of hives has increased from ~530 in 2012 to ~1100 in 2020 (Casanelles‐Abella & Moretti, 2022). Although floral resources probably have not remained constant, it is also unlikely that they have increased at the same pace as urban beekeeping, specifically considering the ongoing densification of Zurich to spare other land covers. With the current biodiversity crisis, in cities known to harbor relatively rich wild bee assemblages (please refer to Casanelles‐Abella, Chauvier, et al., 2021 and Fournier et al., 2020 for Zurich), the effects of livestock raising activities, such as beekeeping, must be not only better understood, but also anticipated. Avoiding high densities of urban beehives, as seen in other cities (e.g., in Paris; Ropars et al., 2019), could allow urban ecosystems to keep preserving wild bees while permitting a certain degree of recreational beekeeping, which can also stimulate engagement in pollinator conservation (Egerer & Kowarik, 2020).

Although our focus was on wild bees, as they are expected to be impacted the most by honeybees, other pollinating insects might also be affected by beekeeping intensity. For example, in Paris, beetle visitation rate was negatively affected by honeybees (Ropars et al., 2019). More studies are required to include other pollinating groups, such as hoverflies, butterflies and beetles (e.g., Dylewski et al., 2019; Ropars et al., 2019). Furthermore, we used available traits deemed important to assess feeding niche overlap. However, future studies could better assess resource‐use overlap by characterizing wild bee feeding behavior more precisely, specifically regarding preferred plant species and nutrient profiles (as done for bumblebees, Kriesell et al., 2017) and plant fidelity. Similarly, measuring the nutritional quality of the studied sites or land covers (Tew et al., 2021), rather than relying on taxonomic metrics, and better accounting for seasonal dynamics, could also help to identify areas where wild bees and honeybees could interact and compete more intensively.

## CONCLUSIONS

Our results indicate a major role of resource availability in driving both the density of honeybees and wild bee species richness patterns, and ultimately in driving feeding niche partitioning between wild bees and honeybees at the community level. In that regard, cities could engage in schemes to monitor resource availability spatially and temporally, as it fluctuates seasonally and depending on weather conditions and urban planning decisions. In addition, because the responses to changes in resource availability and beekeeping intensity might have a lag period, cities should also monitor wild bee populations to better assess the temporal trends and legacies in wild bee populations. These monitoring schemes could help in the planning and regulation of urban beekeeping, for example, guiding where and where not to perform beekeeping, and could promote actions to enhance floral resources to safeguard urban pollinators.

## CONFLICT OF INTEREST

The authors declare no conflict of interest.

## Supporting information


Appendix S1

## Data Availability

Raw and processed data (Casanelles‐Abella, Fontana, et al. 2021) are available from the EnviDAT repository at https://doi.org/10.16904/envidat.253.
