# Supplementary material for: Low resource availability drives feeding niche partitioning between wild bees and honeybees in a European city
Source: Ecol Appl. 2022 Oct 11;33(1):e2727. doi: 10.1002/eap.2727 (PMC10077915; doi:10.1002/eap.2727)
Supplement: Supplementary file 1 — Appendix S1 [file EAP-33-0-s001.pdf]

**Appendix for the paper " Low resource availability drives feeding niche partitioning between wild bees and honeybees in a European city"**

Joan Casanelles-Abella, Simone Fontana, Bertrand Fournier, David Frey and Marco Moretti

**Supplementary Text**

Section S1. Acquisition, sowing and growing of plants

Section S2. Bee traits: rationales and measurement

Section S3. Resource availability at the landscape scale

**Supplementary Tables**

Table S1. Information about the study sites

Table S2. Description bee traits

Table S3. Alternative SEM models

**Supplementary Figures**

Figure S1. Experimental set-up

Figure S2. Metric description

Figure S3. Bee traits

Figure S4. Correlations among response variables and explanatory variables included in the SEM.

Figure S5 Correlations among response variables and explanatory variables included in the SEM.

Figure S6. SEM assumptions

Figure S7. Wild bee community composition in relation to plant species richness

Figure S8. Wild bee community composition in relation to the number of honeybees

Figure S9. Wild bee community composition in relation to the proportion of green surfaces in a 100 m radius

Figure S10. Wild bee community composition in relation to the proportion of green surfaces in a 500 m radius

Figure S11. Wild bee community composition in relation to the number of honeybee hives in 500 m

Figure S12. Change in the values of the six functional traits in relation to feeding niche partitioning

**Section S1.** Seeds of Raphanus sativus were sown on 9 March 2016 into 1.5 L pots, which were filled with commercial standard garden soil and placed in a greenhouse. They were transferred to 7.5 L pots on 22 May. The remaining three species were bought as potted plants from certified Swiss wildflower nurseries (P. Willi, 6274 Eschenbach and UFA Samen, 8408 Winterthur) in March 2016. They were transferred into 10 L pots between 20 and 25 April. Sainfoin plants were grown together in one 20 L pot due to their relatively small size. All plants were kept outdoors under cool conditions from the end of March onwards, in order to harden them. All potted plants were transferred to focal gardens on the same day (9 June) around the onset of flowering. In the gardens, the plants were watered at least weekly and more frequently, based on actual signs of wilting, if necessary.

**Section S2.** ITD is a proxy for body size, which is a measure of mobility and thus foraging distance (Cote et al. 2017).While larger bees (i.e. with a larger ITD) can fly longer distances and consequently could be able to switch foraging patches in case of increased competition (Wojcik et al. 2018; see also Walter-Hellwig et al. 2006), they also have higher nutritional requirements to fuel their flights (Zurbuchen et al. 2010) and may have similar nutrient profile preferences to honeybees (Kriesell et al. 2017). Tongue length is another important functional trait, which determines the range of floral resources (in terms of floral morphology) a bee can access. Specifically, longer tongues enable bees to exploit deeper corollas. Feeding specialization degree is a trait related to the number of plant taxa (i.e. family, genus or species level) a bee forages. We classified bees into three categories: polylectic (visiting >10 plant families), oligolectic (visiting 2–10 plant families, usually few species) and monolectic (visiting 1 plant family, usually one or very few species). Phenology start and end are two traits related to the temporal feeding niche, specifically measuring the amount of time (here weeks) during which a bee is mobile. Finally, we used daily activity (the time of day when a bee was actively foraging) as an additional component of the temporal feeding niche. This trait has been found to be an important aspect of honeybee foraging behaviour (Moore et al. 1989).

ITD was measured following Kendall et al. (2019). Tongue length was measured as the sum of the lengths of prementum and glossa with an Olympus SZX12 Microscope and Olympus image analysis software (Version 510; Olympus Soft Imaging Solutions GmbH, Münster, Germany). Relative tongue length was then calculated by dividing tongue length by ITD to obtain a measure independent of body size (i.e. length of the tongue per unit body size). Daily activity was defined as the time window (1 h intervals) when the bee individual was captured

while feeding. Species-level measurements for the remaining traits were extracted from the European trait database (compiler: Stuart Roberts; pollinator loss module of the EU- FP6 ALARM-project) following Fournier et al. (2020).

**Section S3.** We used a detailed map of the habitats and land-cover types of the city of Zurich (Biotoptypenkartierung der Stadt Zürich 2010), grouped by Braaker et al. (2014) into 11 main categories. In particular, the impervious land-covers include buildings, major roads and gravel. In addition, the green land-covers include urban agriculture (i.e. allotment gardens), meadows, forests, hedges, wetlands and lawn. At each site, we calculated the amount of each category at 50, 100, 250 and 500 m radii. We additionally aggregated the impervious land-covers (i.e. buildings, major roads and gravel) and the green land-covers (agricultural land, meadows, forests, hedges, wetlands and lawn) at the same radii.

**Table S1. Features of the experimental gardens**. For each garden (Site), we provide the geolocation, the number of sampling days, the total sampling effort (i.e. the total amount of time volunteers spend sampling the phytometer), the number of wild bee species, plants species, the abundance of honeybee individuals. Moreover, we provide the amount of green surface around garden at 100 and 500 m radii. Note that a sampling day is equivalent to 9 hours.

| Site | Latitude | Longitude | Sampling days | Sampling effort (days) | Wild bee S | Honeybee abundance | N. hives 500 m | Plant S | Green surfaces 100 m | Green surfaces 500 m |
|---|---|---|---|---|---|---|---|---|---|---|
| 2 | 47.366 | 8.567 | 3 | 5.890 | 27 | 10 | 1 | 148 | 0.5768 | 0.4274 |
| 7 | 47.412 | 8.550 | 5 | 5.330 | 14 | 13 | 0 | 69 | 0.1851 | 0.7771 |
| 8 | 47.411 | 8.565 | 4 | 5.740 | 20 | 28 | 0 | 79 | 0.5919 | 0.5336 |
| 11 | 47.386 | 8.479 | 3 | 6.000 | 15 | 15 | 6 | 89 | 0.5226 | 0.4912 |
| 17 | 47.331 | 8.515 | 3 | 4.810 | 18 | 5 | 0 | 71 | 0.5871 | 0.2957 |
| 19 | 47.371 | 8.540 | 4 | 6.780 | 19 | 85 | 17 | 114 | 0.0385 | 0.9542 |
| 25 | 47.418 | 8.513 | 5 | 6.310 | 28 | 13 | 2 | 132 | 0.6544 | 0.3364 |
| 27 | 47.372 | 8.524 | 3 | 8.000 | 21 | 39 | 20 | 117 | 0.0489 | 0.8891 |
| 28 | 47.365 | 8.518 | 3 | 5.000 | 20 | 27 | 10 | 110 | 0.5132 | 0.7276 |
| 30 | 47.411 | 8.549 | 5 | 5.480 | 28 | 11 | 0 | 185 | 0.343 | 0.8051 |

| | | | | | | | | | | |
|---|---|---|---|---|---|---|---|---|---|---|
| 40 | 47.395 | 8.535 | 4 | 5.370 | 22 | 17 | 10 | 105 | 0.5253 | 0.586 |
| 41 | 47.404 | 8.541 | 4 | 5.560 | 25 | 47 | 0 | 131 | 0.5716 | 0.5175 |
| 47 | 47.407 | 8.577 | 4 | 6.460 | 28 | 24 | 0 | 186 | 0.5596 | 0.5083 |
| 48 | 47.371 | 8.546 | 4 | 7.560 | 15 | 31 | 7 | 102 | 0.1272 | 0.8585 |
| 52 | 47.375 | 8.490 | 4 | 5.520 | 15 | 24 | 3 | 174 | 0.4205 | 0.5406 |
| 53 | 47.375 | 8.523 | 3 | 7.000 | 18 | 46 | 21 | 125 | 0.0953 | 0.8974 |
| 56 | 47.416 | 8.525 | 5 | 5.310 | 23 | 8 | 4 | 171 | 0.7817 | 0.2961 |
| 59 | 47.409 | 8.573 | 4 | 6.190 | 18 | 24 | 0 | 75 | 0.5824 | 0.4937 |
| 71 | 47.352 | 8.582 | 4 | 5.650 | 29 | 15 | 5 | 177 | 0.7604 | 0.2754 |
| 73 | 47.384 | 8.499 | 3 | 6.000 | 20 | 28 | 0 | 76 | 0.3046 | 0.7802 |
| 83 | 47.402 | 8.504 | 4 | 5.000 | 21 | 21 | 17 | 90 | 0.5793 | 0.4248 |
| 84 | 47.385 | 8.576 | 4 | 6.370 | 29 | 31 | 1 | 85 | 0.4575 | 0.3293 |
| 85 | 47.391 | 8.551 | 3 | 5.930 | 22 | 7 | 26 | 82 | 0.7124 | 0.2769 |

**Table S2. Description of the six functional traits used in the analyses.** For each trait, we provide the level of measurement (individual or species), the type of trait, the unit of measurement (for numeric traits) and the categories used (for categorical traits), and a short description.

| **Trait** | **Level of measurement** | **Type** | **Unit** | **Information** |
|---|---|---|---|---|
| Intertegular distance (ITD) | Individual level | Numeric continuous | mm | Reflects body size and thus is a proxy for dispersal and foraging capacity |
| Relative tongue length | Individual level | Numeric continuous | mm | Indicates the diversity of floral morphologies, and consequently resources, a species can access |
| Daily activity | Individual level | Numeric continuous | Hour | Time of the day when a bee species was observed foraging |
| Phenology start | Species level | Numeric discrete | Week | First week of a bee species' active period |
| Phenology end | Species level | Numeric discrete | Week | Last week of a bee species' active period |
| Feeding specialization | Species level | Categorical | Monolectic<br>Oligolectic<br>Polylectic | Classification based on the number of plant taxa visited by a bee species, reflecting bee diet specialization |

**Table S4.** Selected and alternative Structural Equation Models (SEMs). For each SEM, the formulation of the linear model (LM) of the community niche partitioning, wild bee species richness, plant species richness and number of honeybees is presented. For each SM, we report the Akaike Information Criterion (AIC) and corrected Akaike Information Criterion (AICc), the Bayesian Information Criterion (BIC), the adjusted $R^2$ for the four LMs included in the SEM, and the Fisher's C value. Plant S = Plant species richness; MI = management intensity; N honeybees = number of honeybee individuals; Wild bee S = number of wild bee species; Green100 = amount of green surface in 100 m; Green500 = amount of green surface in 500 m; Year = years managed; Hives500 = number of hives in 500 m radius; Agriculture100 = amount of agricultural land in 100 m radius

| | **AIC** | **AICc** | **BIC** | **$R^2$ Niche partitioning** | **$R^2$ Wild bee S** | **$R^2$ Plant S** | **$R^2$ N honeybees** | **Fisher's C** |
|---|---|---|---|---|---|---|---|---|
| **Full model**<br>*Niche partitioning* ~ Plant S + Green500 + N honeybees + Wild bee S + Hives500<br>*Wild bee S* ~ Plant S + N honeybees + Grey100 + Hives500 + Agriculture100<br>*Plant S* ~ MI + Year<br>*N honeybees* ~ Grey500 + Plant S | 83.83 | Inf | 108.81 | 0.54 | 0.57 | 0.39 | 040 | 39.83, df=32, p-value=0.161 |
| **Model 1**<br>*Niche partitioning* ~ Plant S + Green500 + N honeybees + Wild bee S + Hives500<br>*Wild bee S* ~ Plant S + N honeybees + Green100 + Hives500<br>*Plant S* ~ MI + Year<br>*N honeybees* ~ Green500 | 74.52 | 856.99 | 97.23 | 0.54 | 0.44 | 0.39 | 0.40 | 34.52, df=28, p-value=0.184 |
| **Model 2**<br>*Niche partitioning* ~ Plant S + Green500 + N honeybees + Wild bee S + Hives500<br>*Wild bee S* ~ Plant S + N honeybees + Green100 + Hives500 + Agriculture100<br>*Plant S* ~ MI + Year<br>*N honeybees* ~ Green500 + Plant S | 82.89 | 1906.53 | 106.74 | 0.54 | 0.57 | 0.40 | 0.39 | 40.83, df=34, p-value=0.194 |

| | | | | | | | | |
|---|---|---|---|---|---|---|---|---|
| **Model 3**<br>*Niche partitioning* ~ Plant S + Green500 + N honeybees + Wild bee S + Hives500<br>*Wild bee S* ~ Plant S + N honeybees + Green100 + Hives500<br>*Plant S* ~ MI<br>*N honeybees* ~ Green500 + Plant S | 60.76 | 698.72 | 83.47 | 0.55 | 0.54 | 0.40 | 0.357 | 27.72, df=20, p-value=0.116 |
| **Model 4 selected**<br>*Niche partitioning* ~ Plant S + Green500 + N honeybees + Wild bee S + Hives500<br>*Wild bee S* ~ Plant S + N honeybees + Green100 + Hives500<br>*Plant S* ~ MI<br>*N honeybees* ~ Green500 + Plant S | 66.69 | 511.29 | 88.27 | 0.54 | 0.44 | 0.35 | 0.40 | 28.69, df=22, p-value=0.154 |

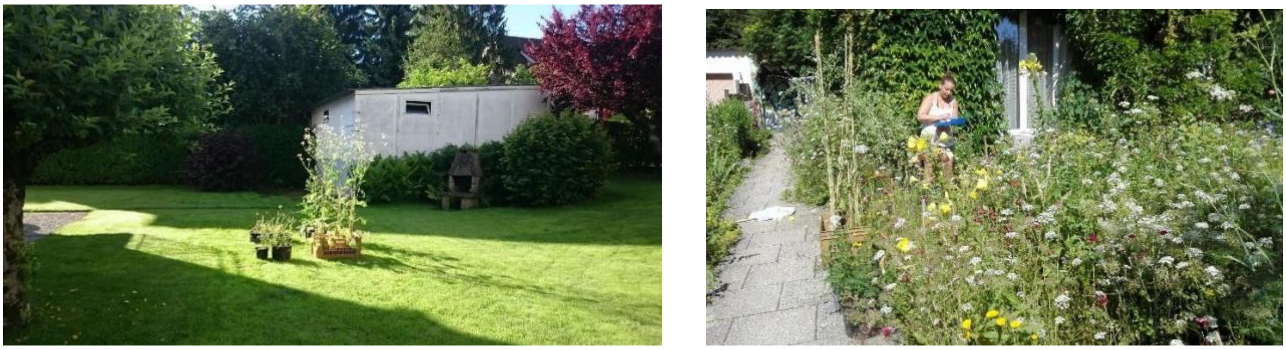

**Figure S1. Experimental set-up.** (a) example of a highly managed garden, with low numbers of plant species, and (b) example of with low management intensity, with high numbers of plant species. Credit: David Frey.

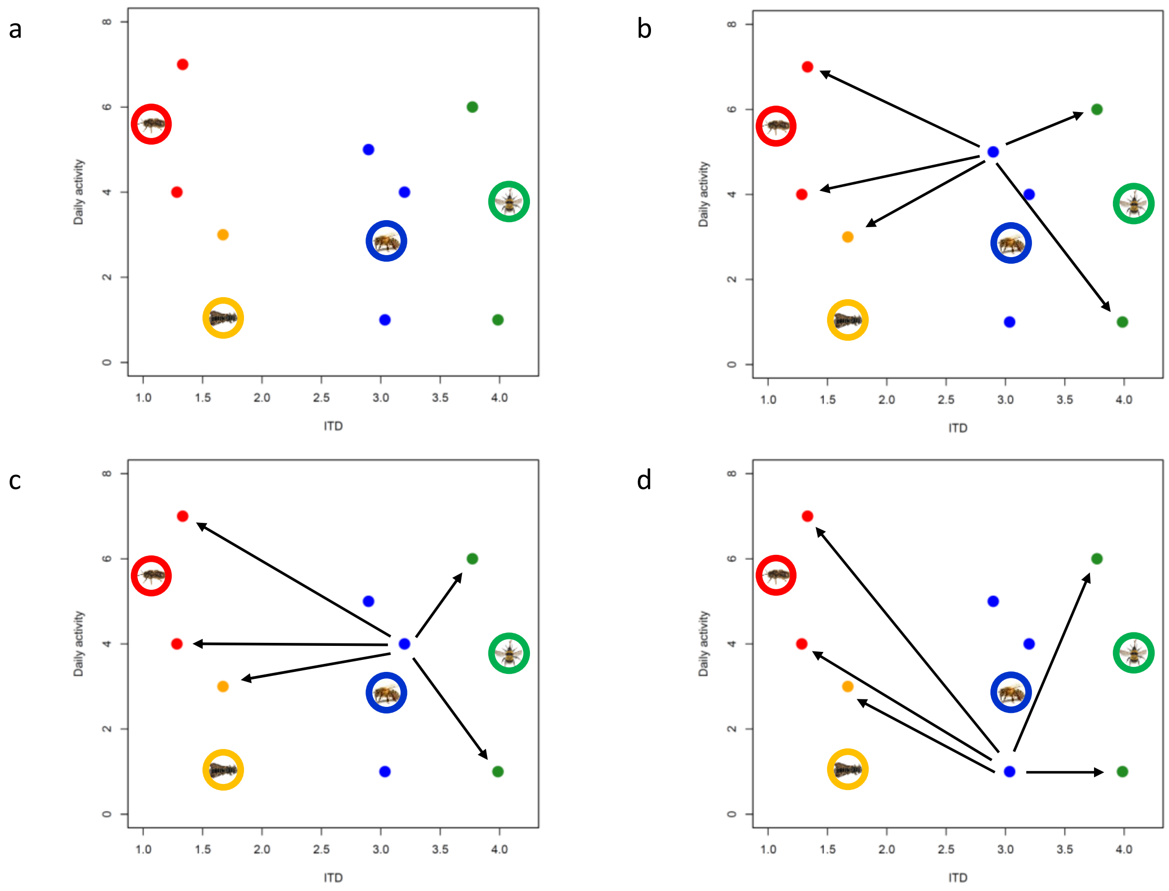


**Figure S2. Graphical summary of the calculation of feeding niche partitioning.** In a given site, feeding niche partitioning between wild bees and honeybees is calculated as the mean of the pairwise distances between all wild bee individuals and all honeybee individuals in the multidimensional trait space (six functional traits in this case, i.e. intertegular distance (ITD), relative tongue length, daily activity, phenology start and end, and feeding specialization). Plots (a–d) show a simplified graphical representation of the calculation considering two traits (two dimensions in the functional trait space, in this case ITD and daily activity), three wild bee species with two, two and one individuals, respectively, and the honeybees with three individuals. Wild bee individuals of the three species are coloured red, yellow and green, and honeybee individuals are coloured blue. Arrows in (b–d) represent the distances between each of the three honeybee individuals and the wild bee individuals of the three species. The average length of the arrows (15 in total in this case) represents feeding niche partitioning.

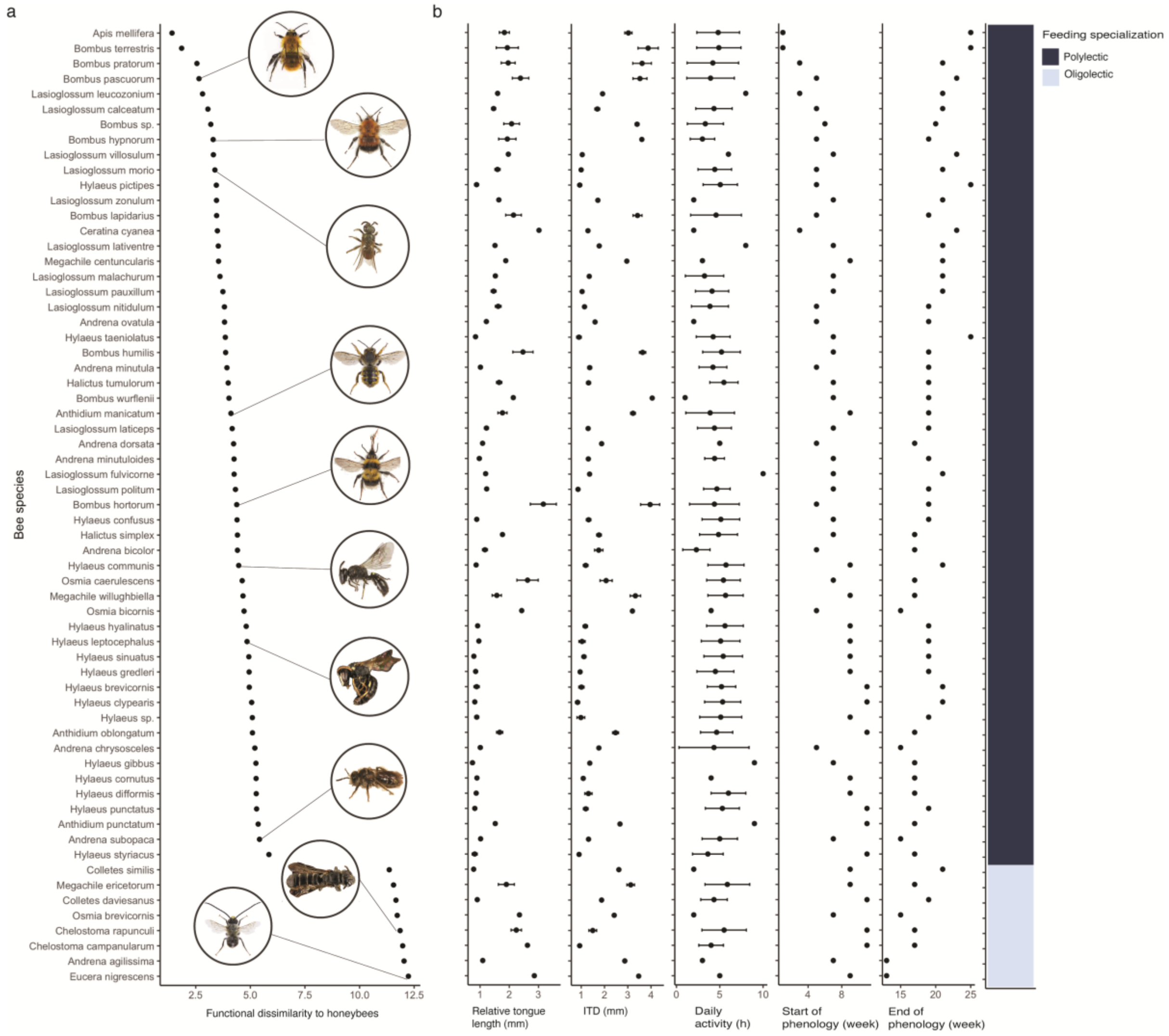


**Figure S3.** Characterization of the studied species. (a) Functional dissimilarity to honeybees, as shown in Figure 2. (b) Values of the six functional traits used to compute the functional dissimilarity per species. For relative tongue length, intertegular distance and day activity, the measurements are at the individual level; dots indicate the mean value per species and bars the standard deviation. The start and end of phenology and feeding specialization (lecty) are measured at the species level. Images retrieved from: https://www.flickr.com/people/usgsbiml/

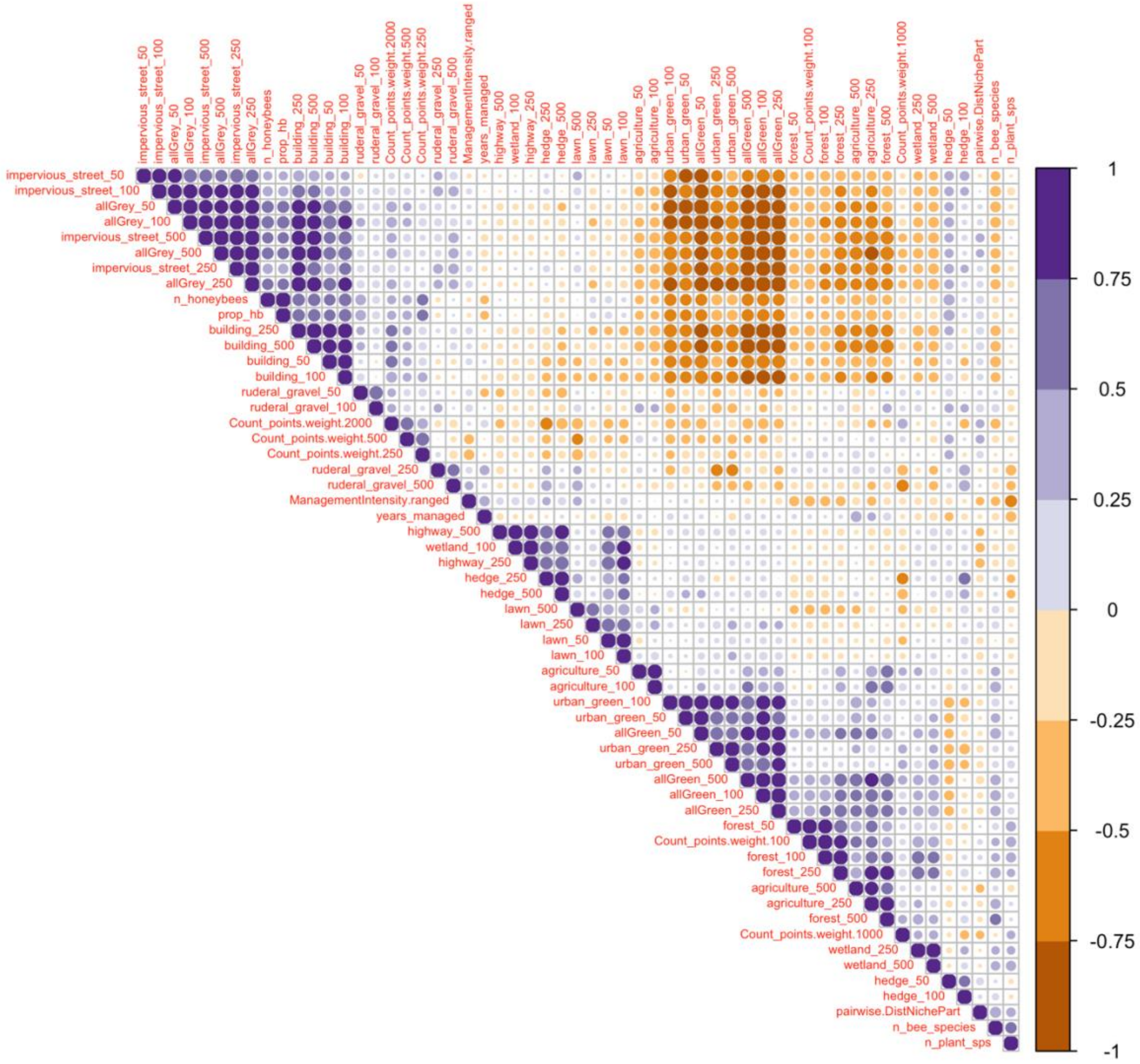


**Figure S4. Correlations among all variables.** ManagementIntensity.ranged = management intensity; n_honeybees = number of honeybee individuals; n_bee_species = number of wild bee species; allGrey50,100,250,500 = amount of grey surface in 50, 100, 250, 500 m; allGrey50,100,250,500 = amount of grey surface in 50, 100, 250, 500 m; Count_poimts.weight.50, .100, .250, .500 = number of hives in 50, 100, 250, 500 m radius; agriculture_50, _100, _250, _500 = amount of agricultural land in 50, 100, 250, 500 m radius; years_managed = years managed

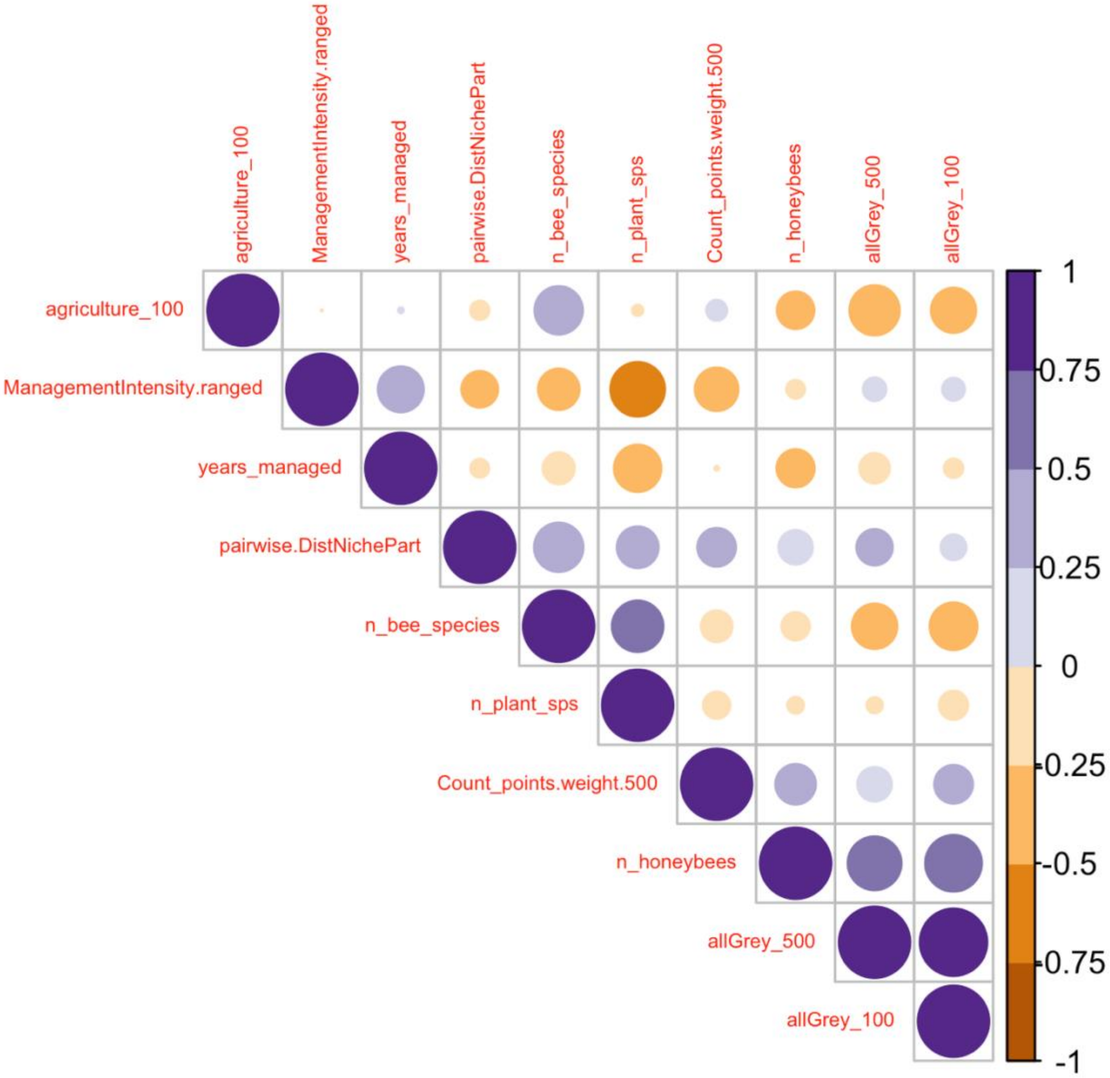


**Figure S5.** Correlations among response variables and explanatory variables included in the SEM. ManagementIntensity.ranged = management intensity; n_honeybees = number of honeybee individuals; n_bee_species = number of wild bee species; allGrey100 = amount of grey surface in 100 m; allGrey500 = amount of grey surface in 500 m; Count_poimts.weight.500 = number of hives in 500 m radius; agriculture_100 = amount of agricultural land in 100 m radius; years_managed = years managed

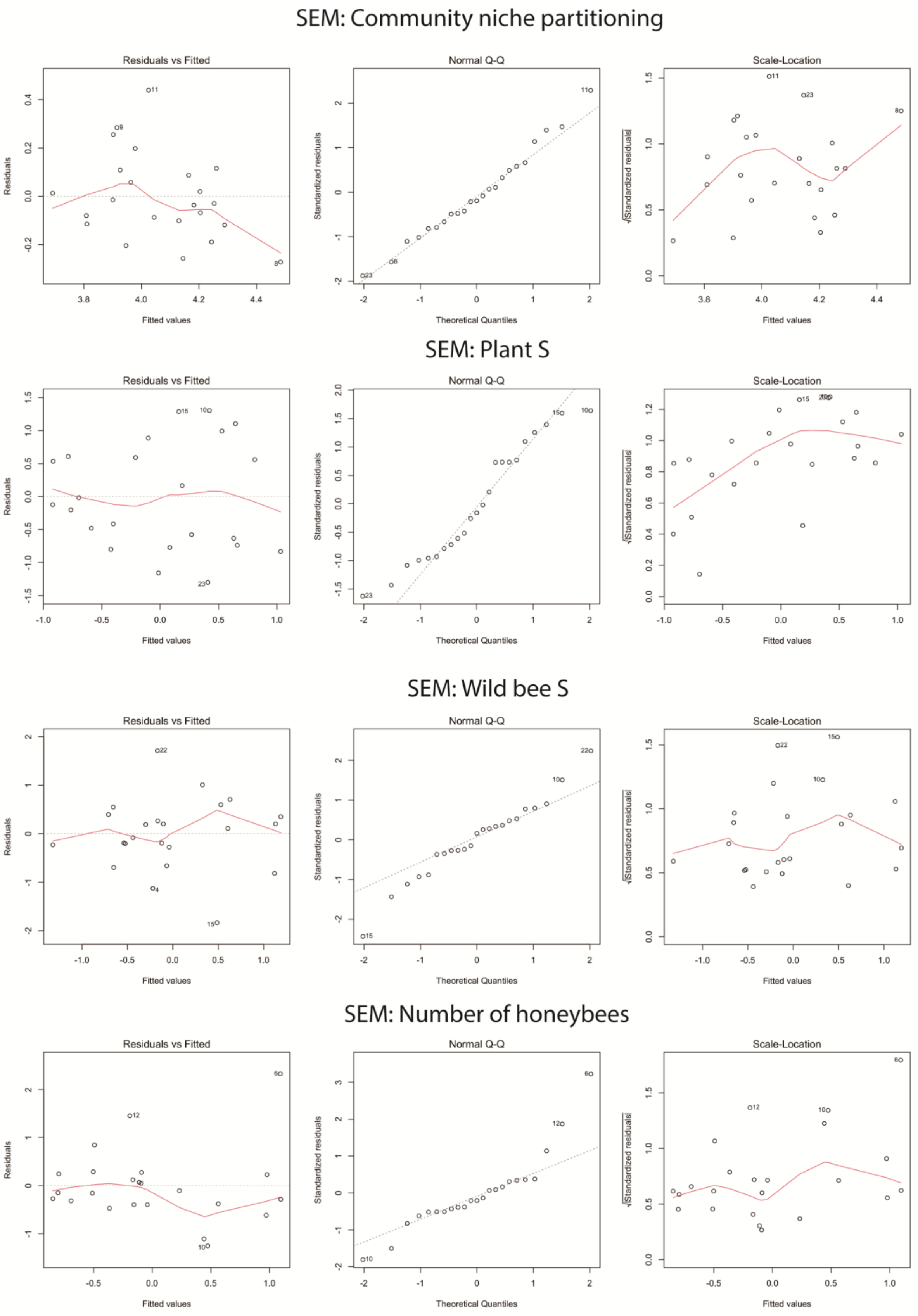


**Figure S6.** Residual plots for assessing model assumptions of the LMs used in the SEM framework. Response variables for the LMs with community niche partitioning, plant species

richness, wild bee species richness and honeybee abundance are plotted (see Table 2 for complete model compositions). Very few measurements do not fit well with the model, as recognizable in the Q-Q plots of the residuals, and the majority of the observations seem to fulfil the model assumptions well. S = species richness

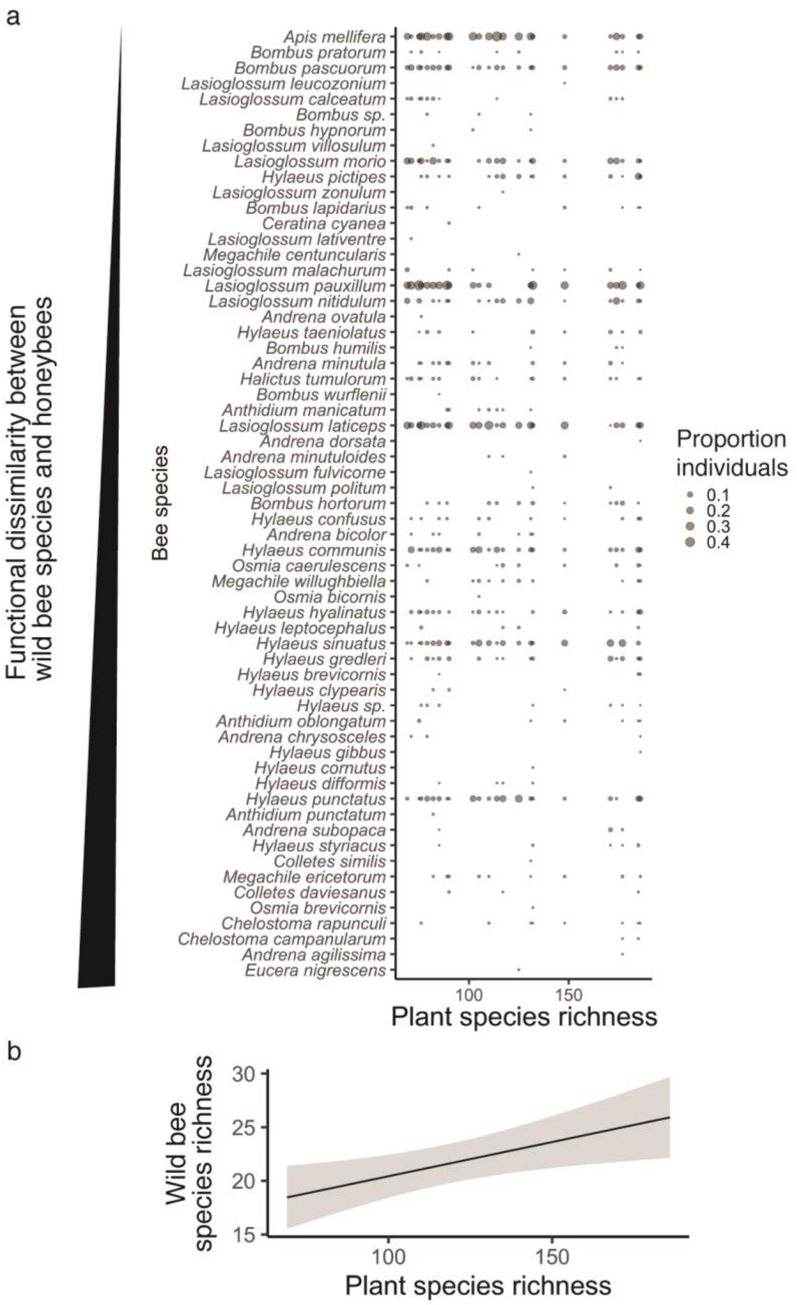


**Figure S7. Influence of resource availability at the local scale on wild bee community composition.** (a) Wild bee species composition in relation to plant richness at each site, which is used a s a proxy of resource availability at the local scale. Wild bee species are sorted according to their functional dissimilarity with honeybees, with functionally similar species on the top and functionally dissimilar species on the bottom. The size of each dot represents the proportion of individuals collected at a given site. (b) Linear model depicting the relationship between the wild bee species richness and plant species richness. The grey band indicates the 95% confidence interval.

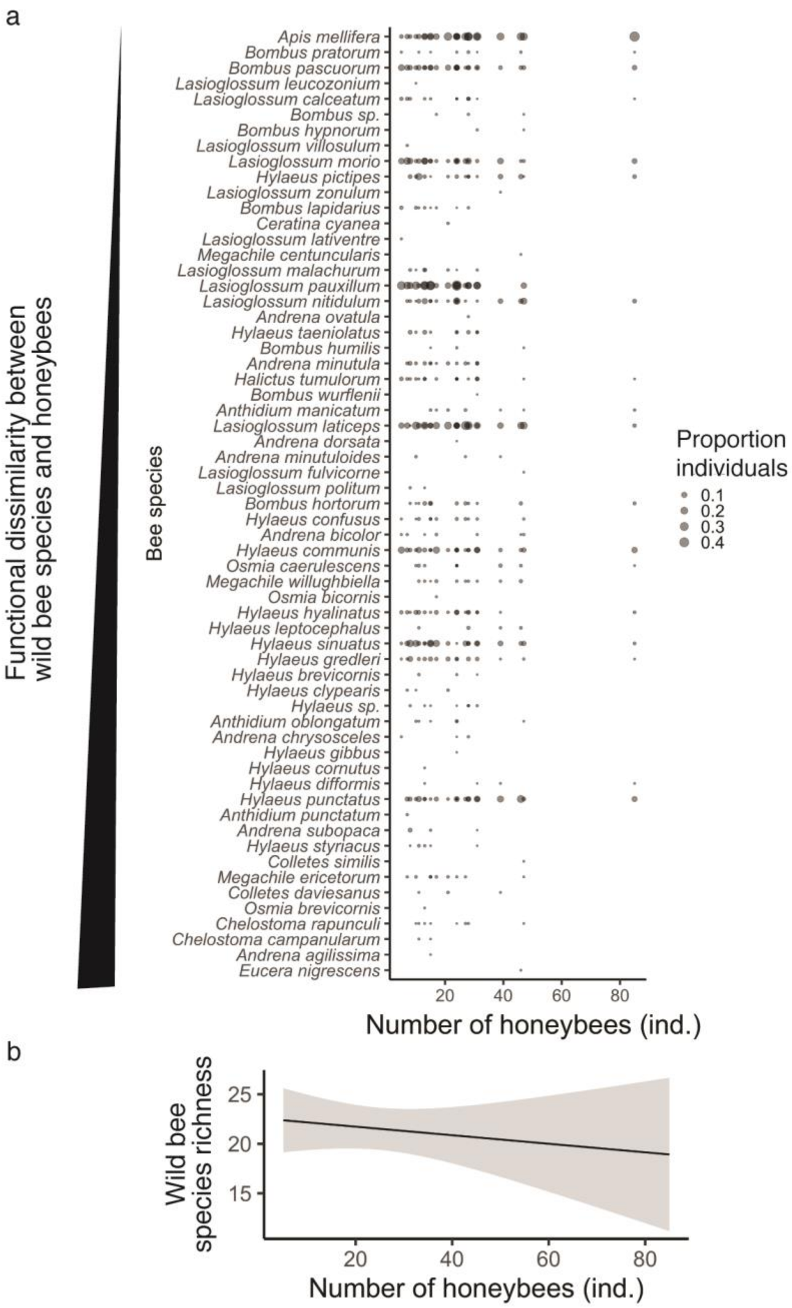


**Figure S8. Influence of beekeeping intensity at the local scale on wild bee community composition.** (a) Wild bee species composition in relation to the number of honeybees at each site, used as a proxy of beekeeping intensity at the local scale. Wild bee species are sorted according to their functional dissimilarity with honeybees, with functionally similar species on the top and functionally dissimilar species on the bottom. The size of each dot represents the proportion of individuals collected at a given site. (b) Linear model depicting the relationship between wild bee species richness and the number of honeybees. The grey band indicates the 95% confidence interval.

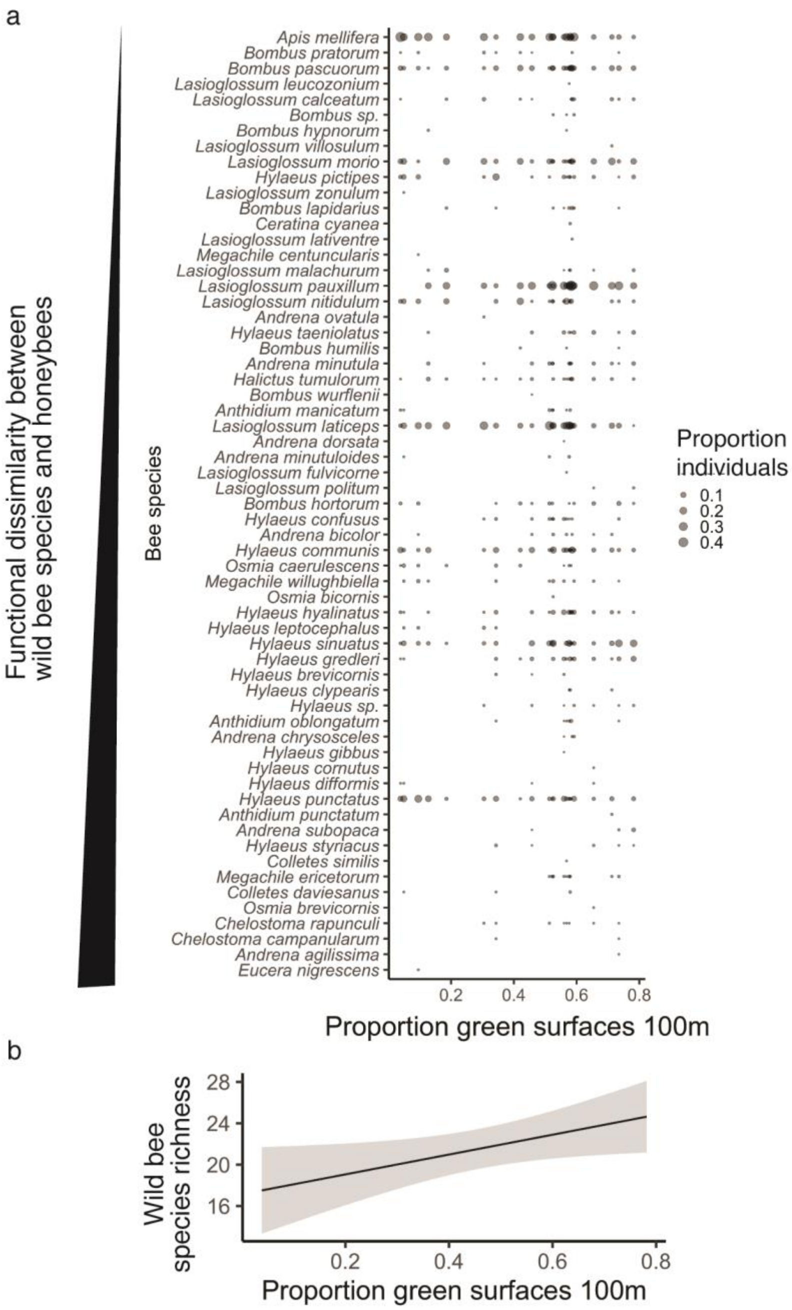


**Figure S9. Influence of the resource availability at the landscape scale on wild bee community composition.** (a) Wild bee species composition in relation to the proportion of grey surfaces in a 100 m radius at each site, used as a proxy of resource availability at the landscape scale. Wild bee species are sorted according to their functional dissimilarity with honeybees, with functionally similar species on the top and functionally dissimilar species on the bottom. The size of each dot represents the proportion of individuals collected at a given site. (b) Linear model depicting the relationship between wild bee species richness and the proportion of green surfaces in a 100 m radius, as also shown in Figure 5d. The grey band indicates the 95% confidence interval.

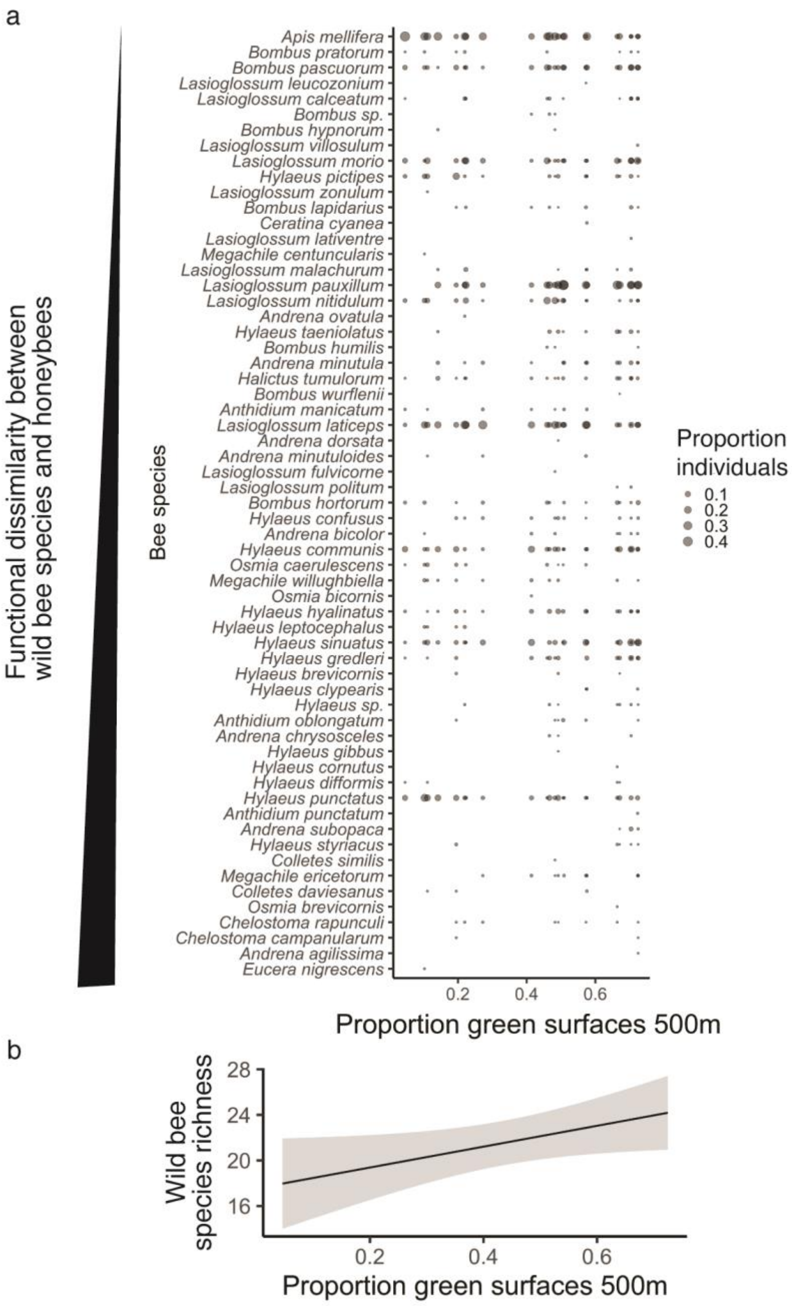


**Figure S10. Influence of the resource availability at the landscape scale on wild bee community composition.** (a) Wild bee species composition in relation to the proportion of green surfaces in a 500 m radius at each site, used as a proxy of resource availability at the landscape scale. Wild bee species are sorted according to their functional dissimilarity with honeybees, with functionally similar species on the top and functionally dissimilar species on the bottom. The size of each dot represents the proportion of individuals collected at a given site. (b) Linear model depicting the relationship between the wild bee species richness and the proportion of green surfaces in a 500 m radius, as also shown in Figure 5b. The grey band indicates the 95% confidence interval.

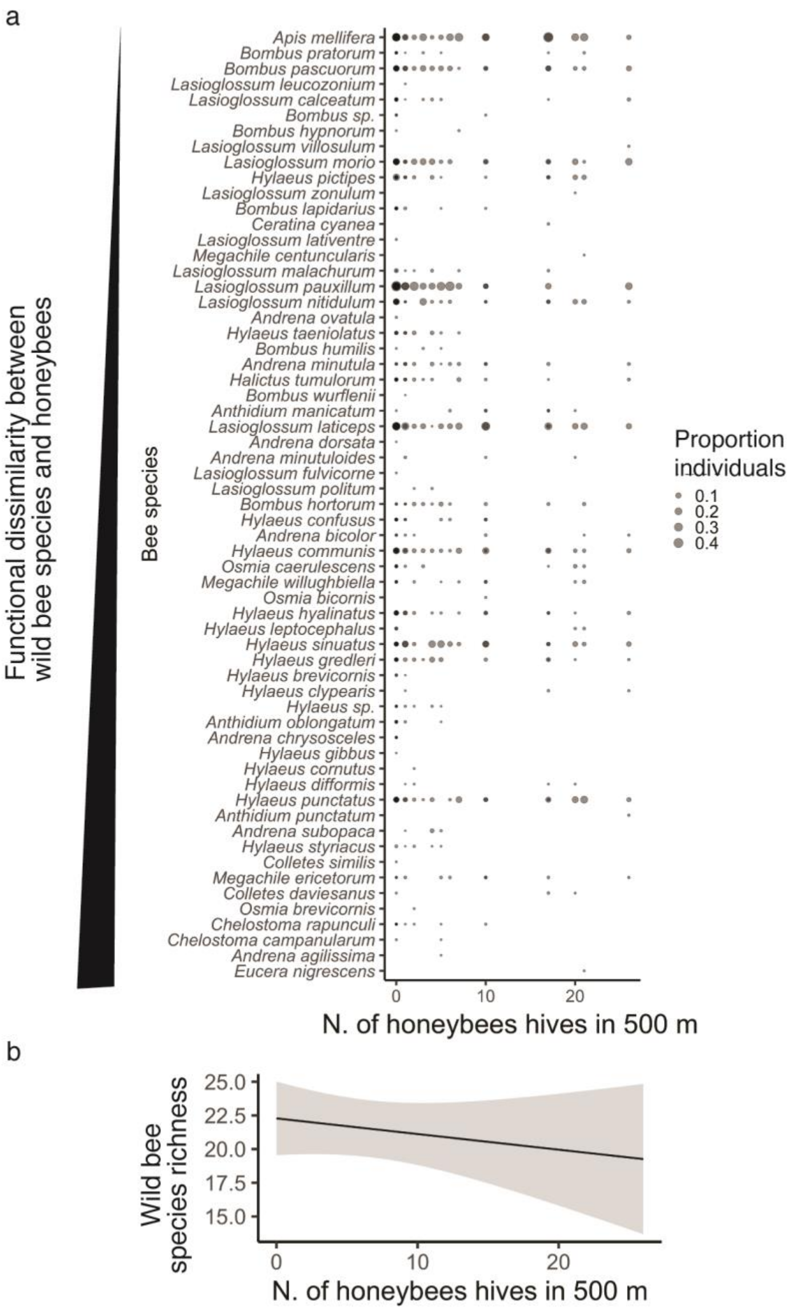


**Figure S11. Influence of beekeeping intensity at the landscape scale on wild bee community composition.** (a) Wild bee species composition in relation to the number of honeybees hives in 500 m, used as a proxy of beekeeping intensity at the landscape scale. Wild bee species are sorted according to their functional dissimilarity with honeybees, with functionally similar species on the top and functionally dissimilar species on the bottom. The size of each dot represents the proportion of individuals collected at a given site. (b) Linear model depicting the relationship between wild bee species richness and the number of honeybees. The grey band indicates the 95% confidence interval.

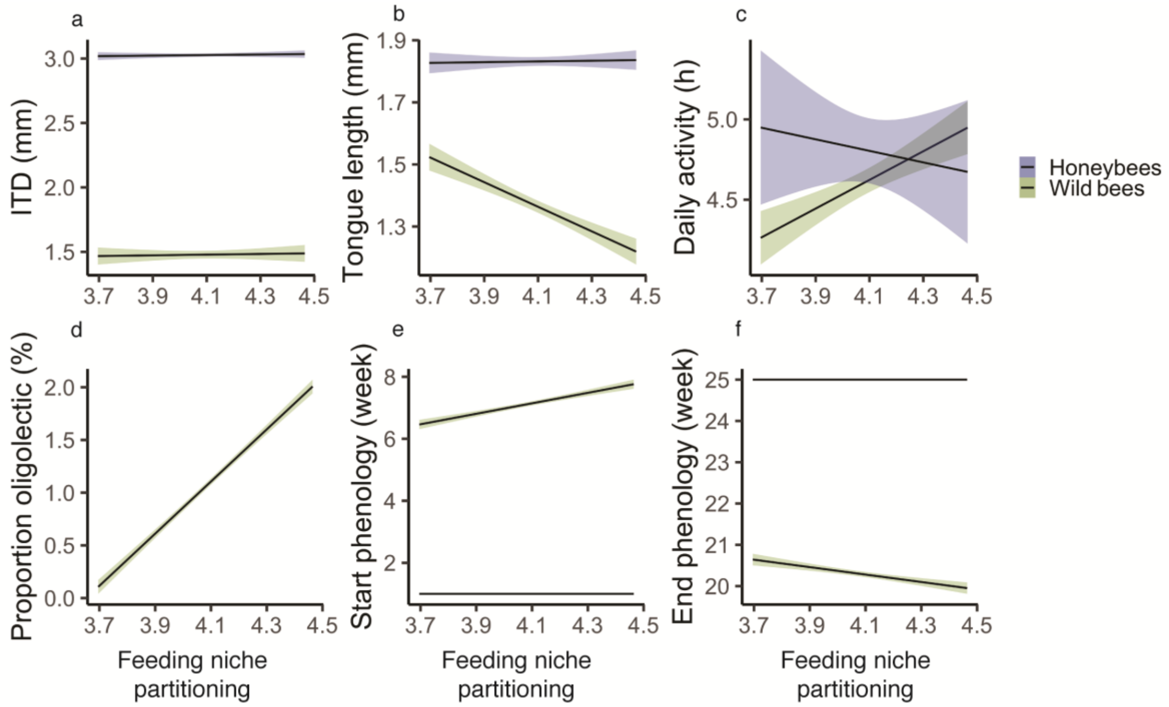


**Figure S12 Change in the values of the six functional traits in relation to feeding niche partitioning.** For each trait, we present the linear model (a, c, d, e, f) or the generalized additive model (b) in relation to feeding niche partitioning for honeybees (green) and wild bees (blue). Green and blue bands represent 95% confidence intervals. Note that for feeding specialization we present the proportion of oligolectic species.

**Supplementary references**


- Biotoptypenkartierung der Stadt Zürich. 2010. Gruen Stadt Zürich (GSZ), Zurich, Switzerland.
- Fournier, B., D. Frey, and M. Moretti. 2020. The origin of urban communities: From the regional species pool to community assemblages in the city. Journal of Biogeography 47:615-629.
- Kendall, L. K., R. Rader, V. Gagic, D. P. Cariveau, M. Albrecht, K. C. R. Baldock, B. M. Freitas, M. Hall, A. Holzschuh, F. P. Molina, J. M. Morten, J. S. Pereira, Z. M. Portman, S. P. M. Roberts, J. Rodriguez, L. Russo, L. Sutter, N. J. Vereecken, and I. Bartomeus. 2019. Pollinator size and its consequences: Robust estimates of body size in pollinating insects. Ecology and Evolution 9:1702–1714.
- Kriesell, L., Hilpert, A. & Leonhardt, S.D. 2017. Different but the same: bumblebee species collect pollen of different plant sources but similar amino acid profiles. Apidologie, 48, 102–116.
- Moore, D., Siegfried, D., Wilson, R. & Rankin, M.A. (1989) The Influence of Time of Day on the Foraging Behavior of the Honeybee, Apis mellifera. Journal of Biological Rhythms, 4:305–325.
- Walther-Hellwig, K., Fokul, G., Frankl, R., Büchler, R., Ekschmitt, K. & Wolters, V. 2006. Increased density of honeybee colonies affects foraging bumblebees. Apidologie 37:517–532.
- Zurbuchen, A., S. Cheesman, J. Klaiber, A. Müller, S. Hein, and S. Dorn. 2010. Long foraging distances impose high costs on offspring production in solitary bees. Journal of Animal Ecology 79:674–681.
